# Vegetarian and Vegan Dietary Patterns to Treat Adult Type 2 Diabetes: A Systematic Review and Meta-Analysis of Randomized Controlled Trials

**DOI:** 10.1016/j.advnut.2024.100294

**Published:** 2024-09-30

**Authors:** Nanci S Guest, Sudha Raj, Matthew J Landry, A Reed Mangels, Roman Pawlak, Katelyn E Senkus, Deepa Handu, Mary Rozga

**Affiliations:** 1 Department of Nutritional Science, Temerty Faculty of Medicine, University of Toronto, Toronto, ON, Canada; 2 Department of Nutrition and Food Studies, David B Falk College of Sport and Human Dynamics, Syracuse University, Syracuse, NY, United States; 3 Program in Public Health, Department of Population Health and Disease Prevention, University of California, Irvine, Irvine, CA, United States; 4 Santa Cruz, CA, United States; 5 Department of Nutrition Science, East Carolina University, Greenville, NC, United States; 6 Department of Human Nutrition, The University of Alabama, Tuscaloosa, AL, United States; 7 Evidence Analysis Center, Academy of Nutrition and Dietetics, Chicago, IL, United States

**Keywords:** vegetarians, vegans, dietary patterns, type 2 diabetes mellitus, systematic review, meta-analysis, randomized controlled trials

## Abstract

Plant-based dietary patterns, including vegetarian and vegan dietary patterns, may help to manage type 2 diabetes (T2DM) by contributing to maintenance of a healthy body weight, improved glycemic control, and reduced risk of diabetes complications. Several diabetes clinical practice guidelines support the use of vegetarian dietary patterns, but there has not been a recently updated systematic review (SR) of evidence from randomized controlled trials (RCTs) to examine efficacy. The primary objective of this SR was to examine the effect of vegetarian dietary patterns compared with nonvegetarian dietary patterns in adults with T2DM. MEDLINE, CINAHL, Cochrane CENTRAL Database of Controlled Trials, Food Science Source, and SportsDiscus databases were searched for RCTs published from 1998 to May 2023. Two independent reviewers extracted data and assessed risk of bias using the Cochrane RoB 2 tool. Data were pooled using a DerSimonian–Laird random-effects model and expressed as mean differences (MDs) with 95% confidence intervals (CIs). Heterogeneity was assessed using the *I*^2^ statistic, and certainty of evidence was assessed using the Grading of Recommendations, Assessment, Development, and Evaluation approach. Full texts of 66 articles were reviewed, and 7 RCTs (*n* = 770 participants) were included in this SR. Vegetarian dietary patterns likely reduce hemoglobin A1c [MD (95% CI): –0.40% (–0.59, –0.21)] and body mass index [MD (95% CI): –0.96 kg/m^2^ (–1.58, –0.34)] (moderate certainty evidence); may allow for reduced diabetes medication (in 2 of 3 included studies) (low certainty); and may improve metabolic clearance of glucose (insulin sensitivity) [MD (95% CI): 10% (1.86, 18.14)] (very low certainty), compared with nonvegetarian dietary patterns. There were no effects of vegetarian dietary patterns on fasting blood glucose, fasting insulin, or low-density lipoprotein cholesterol concentrations. These findings support the inclusion of vegetarian or vegan dietary patterns as options in nutrition care plans for adults with T2DM.

**PROSPERO Registration:**

CRD42023396453.

**Statement of Significance**

Evidence from this systematic review and meta-analysis of 7 randomized controlled trials showed that vegetarian dietary patterns may have benefits for adults with type 2 diabetes including glycemic control, reduced body mass index, and the potential for reduced diabetes medication. However, vegetarian diets may not improve blood low-density lipoprotein cholesterol concentrations.

## Introduction

Diabetes is one of the leading causes of death and disability worldwide, with type 2 diabetes mellitus (T2DM) accounting for ∼95% of diabetes cases [1]. T2DM is commonly associated with obesity, dyslipidemia, and an increased risk of serious health complications such as retinopathy, neuropathy, and nephropathy [2], as well as increased risk of premature death from cardiovascular disease (CVD), cancer, and non-CVD, noncancer causes [3]. Accordingly, there is an urgent need to tackle adverse trends in the prevalence of T2DM risk factors, while also managing the disease in diagnosed individuals.

Diet and lifestyle modifications are key in the management of T2DM and may prevent ≤75% of all cases [4]. Plant-based dietary patterns, including vegetarian and vegan options, may be effective in preventing and managing T2DM by contributing to the maintenance of a healthy body weight, improving glycemic control and reducing the risk of diabetes complications [[5], [6], [7]]. Several prospective cohort studies, such as those based on analysis of the UK Biobank, Adventist and EPIC-Oxford populations, have provided valuable and ongoing insights into the relation between vegetarian dietary patterns and health outcomes, such as T2DM and related comorbidities [[8], [9], [10]].

Diets rich in whole grains, vegetables, fruits, dairy, legumes, and nuts and seeds have been shown to lower the risk of T2DM, whereas those high in sugary drinks and red and processed meat have been linked to an increased risk [[9], [10], [11]]. The 2020–2025 Dietary Guidelines for Americans recommend a Healthy Vegetarian-Style Dietary Pattern, alongside the Healthy Mediterranean-Style Dietary Pattern and the Healthy United States–Style Dietary Pattern, as a dietary option to promote health and prevent disease [12]. Several clinical practice guidelines for diabetes nutrition therapy also support the use of vegetarian dietary patterns, including the European Association for the Study of Diabetes [13], the American Diabetes Association Standard of Care in Diabetes [14], Diabetes UK [15], and Diabetes Canada [16].

Despite the evidence from prospective cohort studies supporting the widespread inclusion of vegetarian dietary patterns in current dietary guidelines and clinical practice guidelines for nutrition therapy in T2DM [11,14,17], there has not been a recently updated systematic review of evidence from randomized controlled trials (RCTs) comparing vegetarian dietary patterns with nonvegetarian dietary patterns in treating adult T2DM. A systematic review is needed to inform healthcare professionals, particularly registered dietitian nutritionists (referred to hereafter as “dietitians”) in clinical care, because they consider the effectiveness of vegetarian or vegan dietary patterns in their nutrition care plans that aim to manage and mitigate risk factors that may worsen T2DM outcomes as they relate to CVD and other comorbidities. The primary objective of this systematic review and meta-analysis using Grading of Recommendations, Assessment, Development, and Evaluations (GRADE) methods was to determine the effect of vegetarian, compared with nonvegetarian, dietary patterns on outcomes of interest in adults with T2DM. A secondary objective was to compare efficacy of interventions with lacto-ovo vegetarian dietary patterns to vegan dietary patterns in adults with T2DM.

## Methods

### Protocol registration and methods

This systematic review was conducted and reported following the PRISMA guidelines [18], and methods from GRADE working group and the Academy of Nutrition and Dietetics [19,20]. The study was registered a priori at The International Prospective Register of Systematic Reviews (PROSPERO) (CRD42023396453) [21]. This systematic review is part of a larger research project examining the effects of vegetarian dietary patterns in different populations, and focuses on the effect in adults with T2DM. Results were posted on the Academy of Nutrition and Dietetics’ Evidence Analysis Library website (https://andeal.org/veg) on 15 February, 2024 [22].

### Search strategy, eligibility criteria, and selection process

The databases search was conducted by an Information Specialist, who searched MEDLINE, CINAHL, Cochrane CENTRAL Database of Controlled Trials, Food Science Source, and SportsDiscus databases. The search included terms such as “vegetarian,” “vegan,” and “plant-based,” with limits on language (published in the English language), publication date (1998–7 May, 2023), and study design (RCTs). The Information Specialist documented the search in each database and de-duplicated results. Relevant systematic reviews were searched for potentially included articles not identified in the databases search. The full search strategy can be found in Supplemental Table 1.

All included studies examined the therapeutic effect of vegetarian or vegan dietary patterns, compared with nonvegetarian dietary patterns or to each other, on outcomes of interest in adults with T2DM. Vegetarian dietary patterns were defined as those excluding meat, poultry, and seafood, with vegan dietary patterns additionally excluding other animal products such as eggs and dairy products [23]. In this article, the term “vegetarian” dietary patterns refers to all types including lacto-ovo vegetarian and vegan patterns. When authors are referring to a specific type of vegetarian diet (e.g., lacto-ovo vegetarian, vegan), that terminology is used. Therapeutic diets were defined as nutrition interventions prescribed by qualified practitioners for disease treatment [24]. Studies were required to be RCTs with interventions ≥4 wk in duration. Outcomes of interest were prioritized by expert panel members and include glycemic outcomes [hemoglobin A1c (HbA1c), fasting blood glucose (FBG), insulin concentrations, insulin sensitivity], diabetes medication/change, disease incidence (CVD, hypertension, CVD events, kidney disease, retinopathy), mortality, quality of life (QoL), BMI (kg/m^2^), LDL cholesterol concentrations, and adverse events. To clarify the role of vegetarian dietary patterns on lipid profile, the authors also examined effects on total, HDL and non-HDL cholesterol, and apoB, which were added as outcomes after registration. A full description of eligibility criteria can be found in Table 1.

**TABLE 1:** Eligibility criteria for systematic review examining the effect of vegetarian compared with nonvegetarian dietary patterns in adults with type 2 diabetes.

	Inclusion criteria	Exclusion criteria
Population	Human adults ≥18 y	Animal studies Humans <18 y
Health status	Studies targeting or with subgroup analysis of adults with T2DM only	Studies for which there is no analysis of adults with T2DM alone Studies that target participants with limited generalizability such as Eating disorders COPD HIV/AIDS Postbariatric surgery Severe/persistent mental illness Pregnancy
Intervention	Vegetarian, including lacto- and lacto-ovo-vegetarian, and/or vegan dietary patterns and all subgroups. Vegetarian dietary patterns were defined as those restricting meat, poultry, and seafood, with vegan dietary patterns additionally restricting other animal products such as eggs and dairy products [23]. Plant-based dietary patterns are only included if the definition meets the definition of vegetarian	Dietary patterns not meeting vegetarian/vegan definition Flexitarian, pescatarian, semivegetarian dietary patterns Studies that do not define “plant-based” and in which it is not possible to determine if plant-based is being used to mean vegetarian/vegan Samples that combine data for semivegetarians and/or pescatarians with vegetarians
Comparison	Nonvegetarian dietary patterns The comparison may be vegetarian compared with vegan dietary patterns Energy restriction if this was characteristic of a “diabetes intervention diet”	No comparison group The comparison group is an intervention that differs from the vegetarian/vegan diet in more ways than inclusion of meat or other animal products
Outcomes	Health outcomes: Disease incidence CVD, HTN, CV events, kidney disease, retinopathy Mortality Diabetes medication/change Quality of life Adverse events Intermediate outcomes: Glycemic outcomes: HbA1c, FBG, insulin levels, insulin sensitivity Other: BMI, total, LDL and HDL cholesterol concentrations; apoB	Outcomes not specified in inclusion criteria
Study design	Randomized controlled trials, parallel or crossover	Nonrandomized or noncontrolled trials, observational studies, systematic and narrative reviews, commentaries
Study duration	≥4 wk	<4 wk
Sample size	≥10 in each group	<10 in each group
Publication dates	1 January, 1998–7 May, 2023	Before 1998 or after 7 May, 2023
Publication status	Peer-reviewed publications	Gray literature, conference abstracts

Abbreviations: COPD, chronic obstructive pulmonary disease; CV, cardiovascular; CVD, cardiovascular disease; FBG, fasting blood glucose; HbA1c, hemoglobin A1c; T2DM, type 2 diabetes mellitus.

Each title/abstract identified in the databases search was screened independently by 2 reviewers using Rayyan software [25]. Each potentially included title/abstract advanced to full-text review, and each article was independently assessed by 2 reviewers to determine eligibility criteria. Any discrepancies in inclusion between reviewers were settled through consensus or a third reviewer.

### Data collection and risk of bias assessment

A standardized template was created to extract data for each included article. Data were extracted by trained evidence analysts and checked by the project manager and included bibliographic information, participant health status and characteristics, and intervention details (e.g., type of vegetarian dietary pattern, study duration, if the intervention included nutrition counseling or energy restriction, etc.), comparison group (e.g., if the nonvegetarian diet was a therapeutic diet), funding source, and outcomes of interest reported.

For continuous variables, analysts extracted sample size, mean change, and variance for each group (within group change). If within group change was not available, analysts extracted pre- and postintervention mean and variance and calculated within group change for each group [26]. If quantitative results were not reported, corresponding authors were contacted and asked to provide the missing values. If missing values were not provided, study results were described narratively only. If a study reported results for multiple time points, the results closest to the end of the intervention were used in analysis.

Risk of bias was assessed using RoB 2: a revised Cochrane risk-of-bias tool for randomized trials [27]. Each article was assessed by 2 independent reviewers and discrepancies were settled by consensus. The RoB 2 tool assesses risk of bias due to the randomization process, deviations from intended interventions, missing outcome data, measurement in the outcome, and selection of the reported results. Studies are rated as having low risk, some concerns, or high risk of bias [27]. Risk of bias is described in a figure produced with robvis software [28].

### Synthesis of results

When possible, each outcome was analyzed in meta-analysis using the DerSimonian–Laird [29] random-effects model due to heterogeneity in prescribed vegetarian dietary patterns and comparison groups. Mean change (within group differences) for each group was imputed to determine mean difference (MD) [95% confidence interval (CI)] between groups for continuous variables. Meta-analysis results are reported with forest plots. Publication bias was not assessed because there was no outcome reported in ≥10 studies [30]. Heterogeneity was assessed using the *I*^2^ measure. Meta-analyses were conducted and forest plots were created using OpenMeta Analyst and RStudio software [26,31]. Subgroup analyses were specified a priori, including according to type of vegetarian dietary pattern (vegan, any/lacto-ovo vegetarian), comparison group (no intervention/usual care, nonvegetarian therapeutic diet), if nutrition counseling and energy restriction were included in the intervention, and study duration (<3, 3–6, or >6 mo). Impact of subgroups on outcomes were examined using multivariable meta-regression including diet type, comparison group, if nutrition counseling was provided, if energy restriction was prescribed, and study duration. Sensitivity analysis was conducted by considering results from studies with low risk of bias only and by conducting leave-one-out analysis to determine if removing any study markedly impacted results.

For each outcome, evidence was described narratively, and a concise conclusion statement was written to directly answer the research question based on certainty of evidence using GRADE informative statements [32]. Certainty of evidence was determined using the GRADE method and a summary of findings table, which considers study design, number of participants, consistency of findings, effect size, precision of findings, directness of evidence, and other factors when grading evidence. Certainty of evidence is rated as “high,” “moderate,” “low,” or “very low” [20].

## Results

### Study selection and characteristics

**FIGURE 1:**
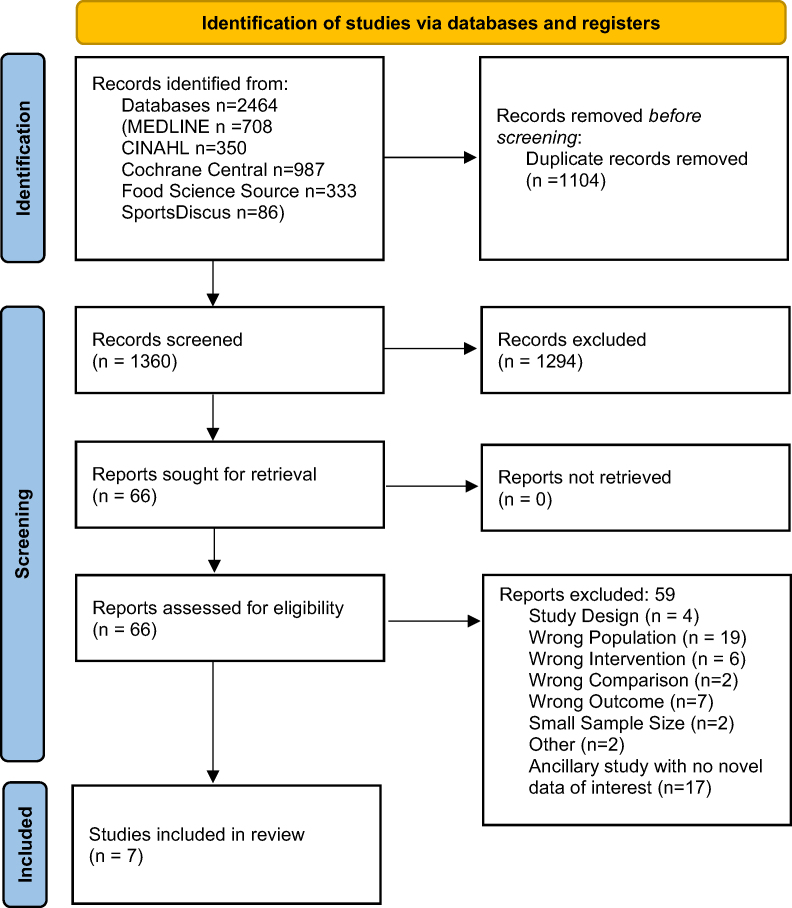
PRISMA flow diagram [18] for systematic review examining the effect of vegetarian dietary patterns in adults with type 2 diabetes.

There were 1360 articles identified in the databases search after de-duplication, full texts of 66 articles were reviewed for inclusion, and 7 RCTs were included in this systematic review (Figure 1). Articles excluded during full text review and reasons can be found in Supplemental Table 2.

**FIGURE 2:**
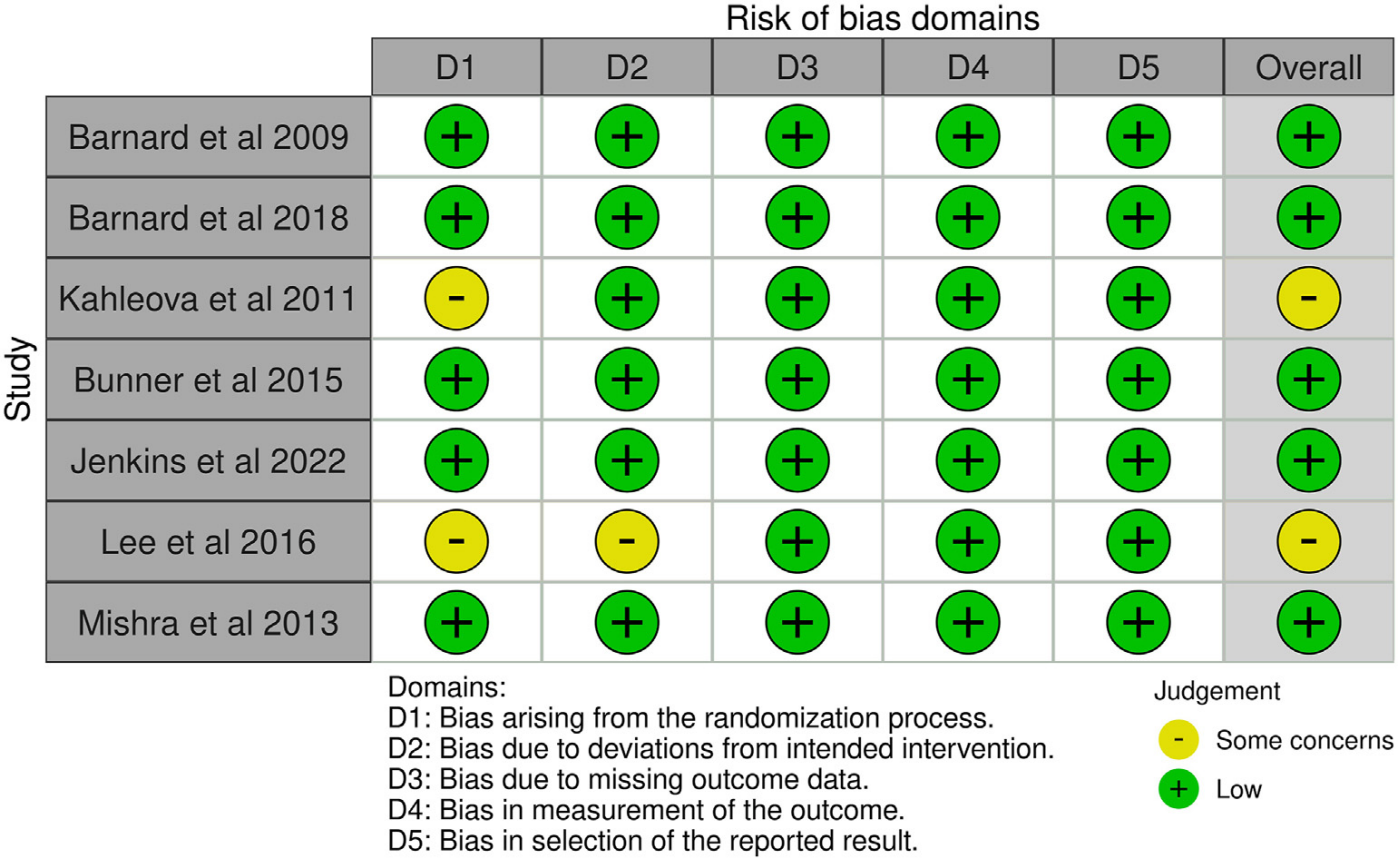
Risk of bias of randomized controlled trials included in a systematic review examining the efficacy of vegetarian dietary patterns in improving outcomes in adults with T2DM. T2DM, type 2 diabetes mellitus.

**FIGURE 3:**
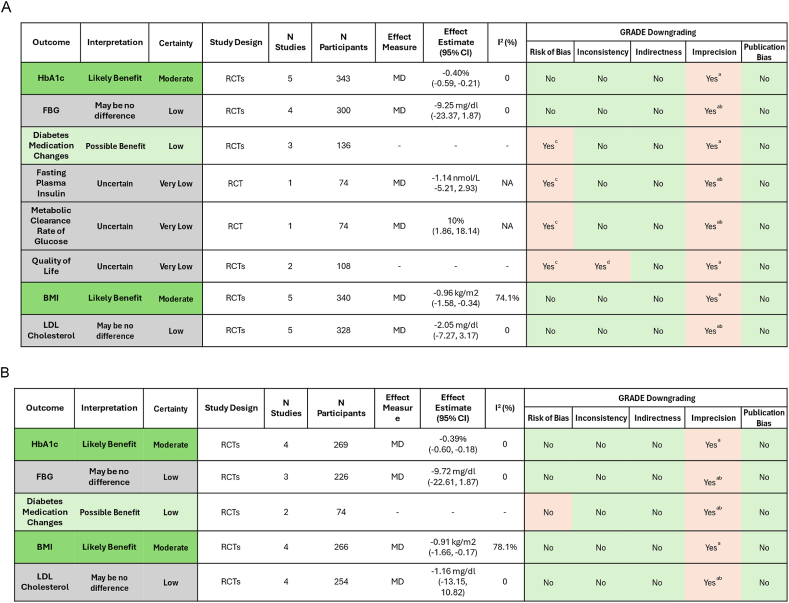
Summary of findings for the systematic review of randomized controlled trials examining the effect of (A) all vegetarian dietary patterns combined and (B) vegan dietary patterns compared with nonvegetarian dietary patterns in adults with type 2 diabetes mellitus. (a) Small sample size, and/or wide confidence interval that may include risk of both benefits and harms. (b) Downgraded 2 levels. (c) Some concerns of risk of bias in included studies. (d) Inconsistency in results between studies. CI, confidence interval; FBG, fasting blood glucose; GRADE, Grading of Recommendations, Assessment, Development, and Evaluation; HbA1c, hemoglobin A1c; MD, mean difference; N, number/sample size; RCT, randomized controlled trial; T2DM, type 2 diabetes mellitus.

Seven RCTs examined the effect of vegetarian dietary patterns on outcomes of interest in adults with T2DM, including 6 RCTs comparing vegetarian with nonvegetarian dietary patterns [[33], [34], [35], [36], [37], [38]] and 1 RCT comparing lacto-ovo vegetarian with vegan dietary patterns [39]. Study and intervention characteristics are shown in TABLE 2, TABLE 3 [[33], [34], [35], [36], [37], [38], [39]]. Risk of bias in included studies is described in Figure 2. Five studies had low risk of bias, and the other 2 studies demonstrated some concerns due to the randomization process [36,37] and deviations from the intended intervention [37]. Details of dietary interventions are shown in Supplemental Table 3. The summary of findings for each outcome is shown in Figure 3. Leave-one-out analysis was conducted for all outcomes for which meta-analysis was conducted. In leave-one-out analyses, there were no appreciable effects on results from removing any one study for the outcomes of HbA1c, BMI, LDL cholesterol, or FBG.

**TABLE 2:** Study characteristics for randomized controlled trials examining the effect of vegetarian or vegan dietary patterns in adults with type 2 diabetes mellitus.

Study or author, year Study design	Country	Target participant health status	Participant mean (SD) age (y) and sex (% F)	Medication	Intervention sample size	Comparison sample size	Outcomes reported	RoB 2 quality	Funder
Barnard et al. 2009 [33] RCT, parallel	United States	T2DM	Intervention: 56.7 (35–82) 55% F Comparison: 54.6 (27–80) 66% F	Remained constant; protocol for endocrinologist to uniformly adjust if needed	49	50	BMI FBG HbA1c LDL-C HDL-C Total-C Adverse events	Low risk	Government Nonprofit
Barnard et al. 2018 [34] RCT, parallel	United States	T2DM	Intervention: 61 (41–79) (mean, range) 62%F Comparison: 61 (30–75) 46% F	Remained constant unless medically necessary	19	21	BMI FGB HbA1c LDL-C HDL-C Total-C	Low risk	Non-profit
Bunner et al. 2015 [35] RCT, parallel	United States	T2DM	Intervention: 57 ± 6 years 65% F Comparison: 58 ± 6 y 44% F	Remained constant unless medically necessary	17	17	BMI FBG HbA1c LDL-C HDL-C Total-C Medication QoL	Low risk	NR
Jenkins et al. 2022 [39] RCT, parallel	Canada	T2DM	Vegan: 59 ± 8.6 54.2% F Vegetarian: 58 ± 11.7 54.3% F	Changes in diabetes, hypertensive, and lipid-lowering medications were outcomes	74	76	BMI HbA1C FBG LDL-C Medication Adverse events	Low	Industry
Kahleova et al. 2011[36] RCT, parallel	Czech Republic	T2DM Overweight or obesity	Intervention: 56.4 ± 7.8 Comparison: 57.7 ± 4.9	Medication kept constant expect with repeated hypoglycemia	31	31	BMI FBG HbA1c Insulin LDL-C HDL-C Total-C QoL	Some concerns	Government
Lee et al. 2016 [37] RCT, parallel	Korea	T2DM	Intervention: 57.5 ± 7.7 y 87%F Comparison: 58.3 ± 7.0 y 74.5% F	Medication kept constant	46	47	FBG HbA1c LDL-C HDL-C	Some concerns	Government
Mishra et al. 2013 [38] RCT, parallel	United States	Overweight or obesity OR previous diagnosis of T2DM (data on HbA1c for T2DM only)	Intervention: 44.3 ± 15.3 y 77% F Comparison: 46.1 ± 13.6 y	Medication remained constant during intervention	142	149	HbA1c	Low risk	Not-for-profit

Abbreviations: BP, blood pressure; CV, cardiovascular; CVD, cardiovascular disease; F, female; FBG, fasting blood glucose; HbA1c, hemoglobin A1c; NR, not reported; QoL, quality of life; RoB 2, version 2 of the Cochrane risk-of-bias tool for randomized trials; T2DM, type 2 diabetes mellitus; Total-C, total cholesterol; TG, triglycerides.

**TABLE 3:** Intervention characteristics for randomized controlled trials examining the effect of vegetarian or vegan dietary patterns in adults with type 2 diabetes mellitus.

Study author, year	Type of intervention diet	Study duration	Comparison diet	Energy restriction	Supplement	Individualized diet counseling	Other interventions beyond nutrition education/counseling	Dietary adherence measured	Dietary assessment method
Barnard et al. 2009 [33]	Vegan, low-fat	74 wk	ADA diet, individualized	Intervention: none Comparison: yes, if overweight	Both groups: vitamin B12	Both groups: RD counseling	Both groups: cooking instruction	Yes	24-h diet recall 3-d diet record
Barnard et al. 2018 [34]	Vegan, low-fat	20 wk	Portion-controlled	Intervention: none Comparison: yes	Both groups: vitamin B12	Both groups: RD counseling	None	Yes	24-h diet recall
Bunner et al. 2015 [35]	Vegan	20 wk	No intervention	None	Both groups: vitamin B12	None	None	Yes	2-d diet records
Jenkins et al. 2022 [39]	Vegan	12 wk	Lacto-ovo-vegetarian	Both groups: yes	None	NR	Food (not meals) provided	Yes	7-d food records
Kahleova et al. 2011 [36]	Lacto-ovo-vegetarian	24 wk	Conventional diabetic diet	Both groups: yes	Both groups: vitamin B12	NR	Both groups: Exercise, meals provided, cooking classes	Yes	Food pick-up; 3-d diet record
Lee et al. 2016 [37]	Vegan	12 wk	Korean Diabetic Association Diet	Intervention: no Comparison: yes	None	Both groups: RD counseling	None	Yes	24-h dietary recall
Mishra et al. 2013 [38]	Vegan, low-fat	18 wk	No intervention	None	Intervention: vitamin B12	None	None	Yes	24-h diet recall

Abbreviations: NR, not reported; RD, registered dietitian.

### Effect of vegetarian dietary patterns on glycemic control

#### Hemoglobin A1c

**FIGURE 4:**
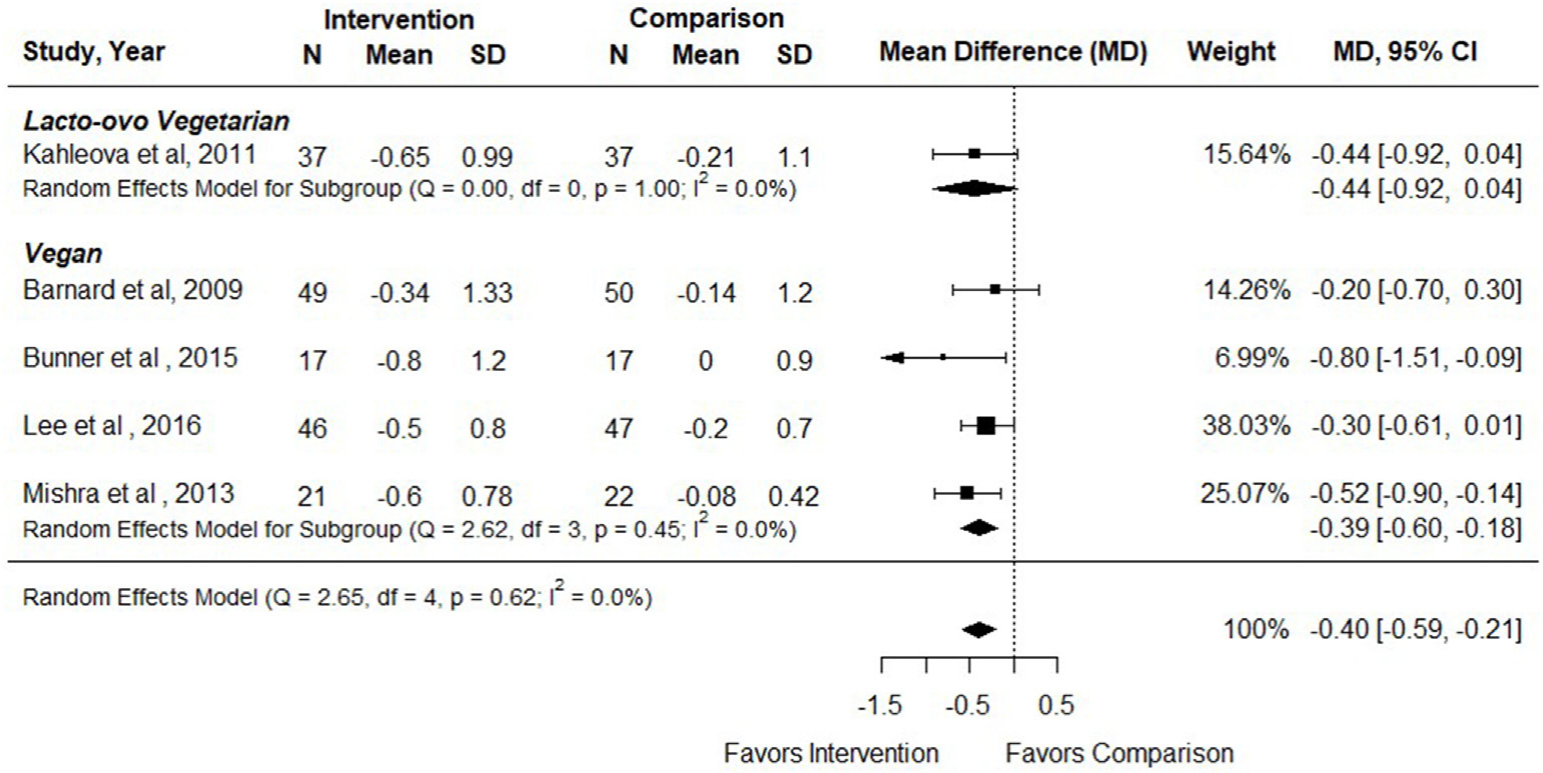
Forest plot of a meta-analysis describing the effect of vegetarian compared with nonvegetarian dietary patterns on HbA1c (%) in adults with type 2 diabetes. CI, confidence interval; MD, mean difference; N, sample size.

Six RCTs examined the effect of vegetarian, compared with nonvegetarian, dietary patterns on HbA1c (%) in adults with T2DM (*n* = 383) [[33], [34], [35], [36], [37], [38]], and all but 1 study [34] reported data that could be included in meta-analysis. Interventions ranged from 12 to 74 wk duration (mean: 25.7 wk). In Mishra et al. [38], only the subgroup of participants with T2DM was included when analyzing the outcome HbA1c. In meta-analysis of 5 studies, there was a greater decrease in HbA1c % in participants assigned vegetarian compared with nonvegetarian dietary patterns [MD (95% CI): –0.40% (–0.59, –0.21); *I*^2^ = 0%]. In subgroup analysis, there was a reduction shown from vegan [–0.39% (–0.60, –0.18); *I*^2^ = 0%], but not lacto-ovo vegetarian [–0.44% (–0.92, 0.04); *I*^2^ = NA], dietary patterns on HbA1c compared with nonvegetarian dietary patterns (Figure 4 and Supplemental Table 4), although there was only 1 study examining a lacto-ovo vegetarian pattern and CIs overlapped. In multivariable meta-regression, there was no impact of any of the examined components on HbA1c% (omnibus *P* = 0.53). Sensitivity analysis revealed that results persisted when only studies with low risk of bias were considered [–0.46% (–0.74, –0.19) (*n* = 176); *I*^2^ = 0.5%]. Barnard et al. [34] was not included in meta-analysis, because results were reported as medians without variance, but authors found no difference in HbA1c % between participants following a low-fat vegan diet compared with a portion-controlled diet (–0.4% in both groups). Evidence certainty was moderate due to small sample sizes (Figure 3). In adults with T2DM, vegetarian and vegan dietary patterns likely reduce HbA1c % compared with therapeutic or nontherapeutic nonvegetarian dietary patterns.

An additional study compared effects of low-carbohydrate vegan and lacto-ovo vegetarian dietary patterns on HbA1c [39]. Jenkins et al. [39] reported a reduction in HbA1c for both dietary treatments after a 12-wk intervention, with no difference between groups [–0.10% (–0.22, 0.02)].

#### Fasting blood glucose

**FIGURE 5:**
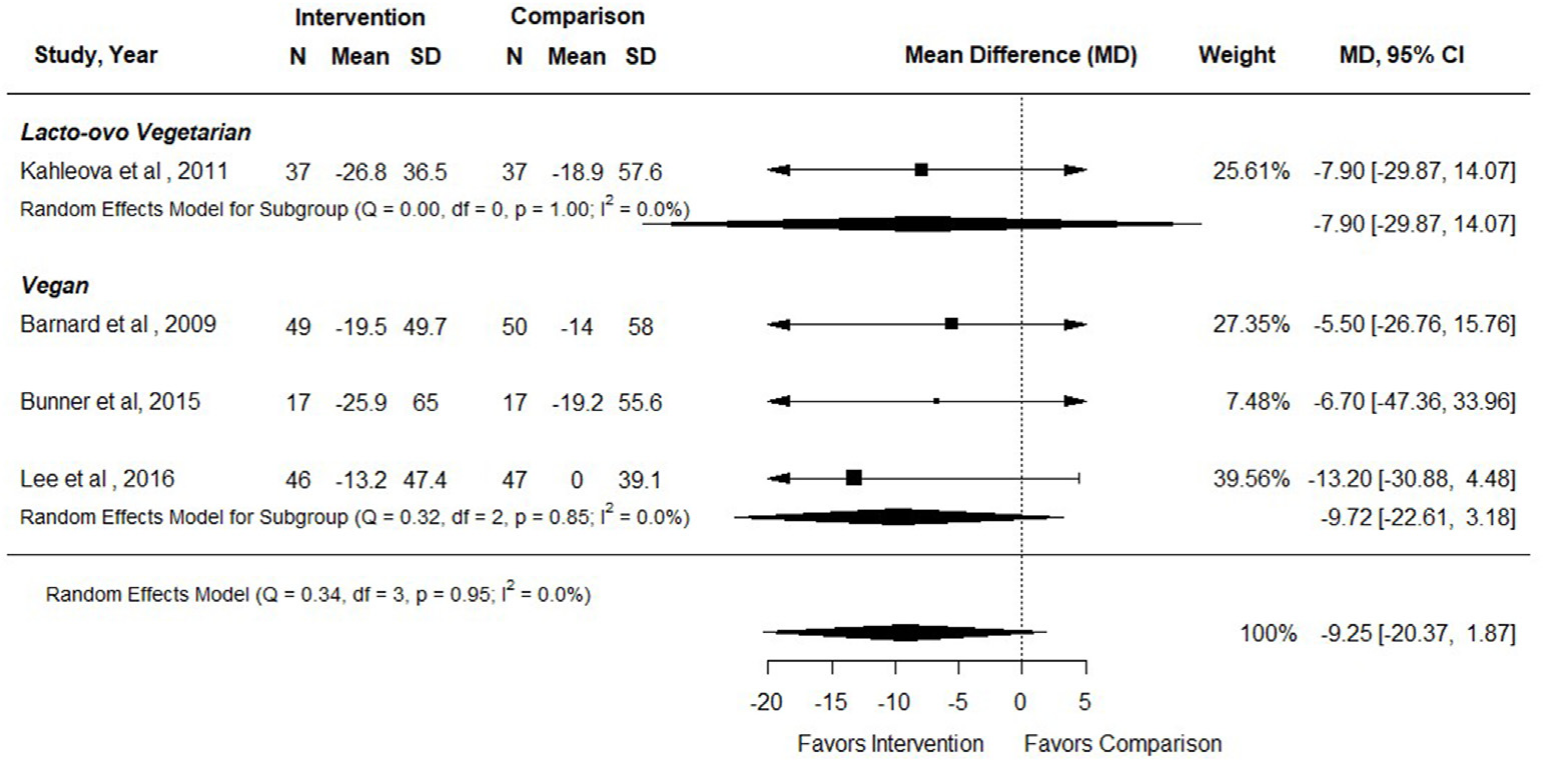
Forest plot of a meta-analysis describing the effect of vegetarian compared with nonvegetarian dietary patterns on fasting blood glucose (mg/dL) in adults with type 2 diabetes. CI, confidence interval; MD, mean difference; N, sample size.

Five RCTs examined the effect of vegetarian, including vegan, dietary patterns compared with nonvegetarian dietary patterns on FBG concentrations in adults with T2DM (*n* = 340) [[33], [34], [35], [36], [37]], and all but 1 study [34] reported data that could be included in the meta-analysis. Intervention durations ranged from 12 to 74 wk. In meta-analysis of 4 RCTs (*n* = 300), there was no difference in FBG concentrations in participants following vegetarian compared with nonvegetarian dietary patterns [MD (95% CI): –9.25 mg/dL (–20.37, 1.87); *I*^2^ = 0%] (Figure 5). In subgroup analysis, there was no impact of either vegan or lacto-ovo vegetarian compared with nonvegetarian dietary patterns on FBG (Figure 5). Sensitivity analysis revealed similar results for studies with low risk of bias only [–5.76 mg/dL (–24.60, 13.08) (*n* = 133); *I*^2^ = 0%]. Barnard et al. [34] was not included in meta-analysis as results were reported as medians without variance, but authors found no difference in FBG concentrations between participants following a low-fat vegan dietary pattern compared with a portion-controlled dietary pattern (median of –16 compared with –12.5 mg/dL, *P* = 0.71). Evidence certainty was low due to small sample sizes and a wide CI including both potential benefits and harms of the dietary intervention (Figure 3). In adults with T2DM, vegetarian, including vegan, dietary patterns may have little or no effect on FBG concentrations compared with therapeutic or nontherapeutic nonvegetarian dietary patterns.

An additional study compared effects of vegan and lacto-ovo vegetarian dietary patterns on FBG concentrations [39]. Jenkins et al. [39] reported a reduction in FBG for both treatment groups after a 12-wk intervention, with no difference in FBG between vegan and vegetarian dietary patterns [0.19 mmol/L (–0.23, 0.61 mmol/L)].

#### Diabetes medication changes

Three RCTs examined the effect of vegetarian, compared with nonvegetarian, dietary patterns on diabetes medication changes in adults with T2DM (*n* = 136) [[34], [35], [36]]. Interventions ranged from 20 to 24 wk. Data were reported using heterogeneous measures and could not be pooled in meta-analysis. Barnard et al. [34] reported that for participants in the vegan group, medication was reduced (19.0%), mixed (9.5%), or could not be accurately assessed (9.5%). In the portion-controlled group, medications were reduced (25%), mixed (16.7%), or increased (12.5%). Bunner et al. [35] reported that in the vegan group, glucose-lowering medications were reduced in 58.8% of participants and increased in 2 participants (11.8%). In the comparison group with no intervention, glucose-lowering medications were reduced in 1 participant (5.9%) and increased in 2 participants (11.8%). In the study by Kahleova et al. [36], 43% of participants following a lacto-ovo vegetarian diet reduced their diabetes medication due to repeated hyperglycemia compared with 5% following a conventional diabetes diet [MD (95% CI): 38% (17–58); *P* < 0.001]. Evidence certainty was low due to risk of bias in included studies and small sample size (Figure 3). In adults with T2DM, evidence on the effect of vegetarian, compared with nonvegetarian, dietary patterns was mixed but suggests some benefit of vegetarian dietary patterns on diabetes medication compared with therapeutic or nontherapeutic nonvegetarian dietary patterns.

One RCT compared changes in diabetes medications between vegan and lacto-ovo vegetarian dietary patterns [39]. Jenkins et al. [39] reported no differences in the change of diabetes medications between groups over the 12-wk intervention.

#### Fasting plasma insulin

One RCT examined the effect of a lacto-ovo vegetarian dietary pattern, compared with a conventional diabetic diet, on fasting plasma insulin in adults with T2DM and overweight or obesity (*n* = 74) [36]. After 24 wk, there was no difference in fasting plasma insulin concentrations compared with a conventional diabetic diet [MD (95% CI): –1.14 nmol/L (–5.21, 2.93)]. Evidence certainty was very low due to risk of bias in the included study, small sample size, and a wide CI crossing including both potential benefits and harms (Figure 3). In adults with T2DM, there may be no difference in fasting plasma insulin between participants following a lacto-ovo vegetarian dietary pattern compared with a therapeutic diet for 24 wk, but evidence is uncertain.

#### Insulin sensitivity

One RCT examined the effect of a lacto-ovo vegetarian dietary pattern, compared with a conventional diabetic diet, and metabolic clearance rate of glucose using a hyperinsulinemic isoglycemic clamp in adults with T2DM and overweight or obesity (*n* = 74) [36]. After 24 wk, there was a greater increase in metabolic clearance rate of glucose in the group following a lacto-ovo vegetarian diet compared with the conventional diabetic diet [MD (95% CI): 10% (1.86, 18.14)]. Evidence certainty was very low due to risk of bias in the included study and very small sample size (Figure 3). In adults with T2DM, lacto-ovo vegetarian dietary patterns may improve metabolic clearance rate of glucose (insulin sensitivity) compared with a therapeutic diet after 24 wk, but evidence is uncertain.

### Effect of vegetarian dietary patterns on QoL

Two RCTs examined the effect of vegetarian, compared with nonvegetarian, dietary patterns on QoL in adults with T2DM (*n* = 108) [35,36]. Kahleova et al. [36] assessed QoL using the Obesity and Weight-Loss Quality-of-Life tool and found a greater improvement in score in the lacto-ovo vegetarian group compared with the conventional diabetic diet group after 24 wk (*P* = 0.01). Bunner et al. [35] utilized the Norfolk Quality of Life Questionnaire and found that score improved in both the vegetarian and control groups at 20 wk, but there was no difference between groups (*P* = 0.43). Evidence certainty was rated as very low due to risk of bias in included studies, inconsistency in results between studies and small sample sizes (Figure 3). In adults with T2DM, the effect of vegetarian, compared with nonvegetarian, dietary patterns on QoL is uncertain.

### Effect of vegetarian dietary patterns on BMI

**FIGURE 6:**
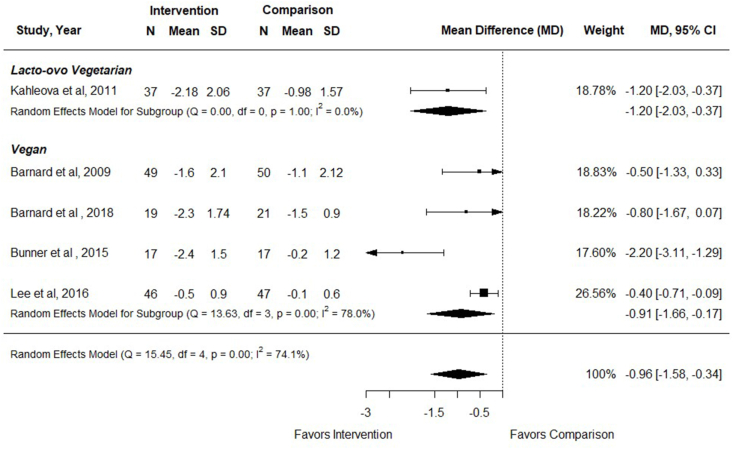
Forest plot of a meta-analysis describing the effect of vegetarian compared with nonvegetarian dietary patterns on BMI (kg/m^2^) in adults with type 2 diabetes. CI, confidence interval; MD, mean difference; N, sample size.

Five RCTs examined the effect of vegetarian, compared with nonvegetarian, dietary patterns on BMI in adults with T2DM (*n* = 340) [[33], [34], [35], [36], [37]], and all studies reported data that could be included in meta-analysis. Interventions ranged from 12 to 74 wk duration. In meta-analysis of 5 studies, participants assigned vegetarian dietary patterns had decreased BMI compared with participants following nonvegetarian dietary patterns [MD (95% CI): −0.96 (–1.58, –0.34); *I*^2^ = 74.1%] (Figure 6). BMI was reduced with both vegan and lacto-ovo vegetarian dietary patterns, although there was only 1 RCT examining a lacto-ovo vegetarian dietary pattern (Figure 6 and Supplemental Table 4). There was a greater reduction in BMI when compared with no intervention than when compared with a nonvegetarian therapeutic diet (Supplemental Table 4), and comparison group explained a large portion of heterogeneity. However, there was only 1 study examining a lacto-ovo vegetarian diet. In multivariable meta-regression, there was an effect of the comparison group such that there was a greater impact of vegetarian dietary patterns compared with no dietary intervention than when compared with a therapeutic dietary intervention (*P* < 0.001). In sensitivity analysis, outcomes were also improved when analyzing studies with low risk of bias only [–1.15 (–2.16, –0.14); *I*^2^ = 75.1%]. Evidence certainty was moderate due to small sample sizes (Figure 3). In adults with T2DM, vegetarian and vegan dietary patterns likely reduce BMI compared with therapeutic or nontherapeutic nonvegetarian dietary patterns.

One additional study compared effects of vegan and lacto-ovo vegetarian dietary patterns on BMI [39]. Jenkins et al. [39] reported a reduction in BMI for both treatment groups after a 12-wk intervention period. There was no difference on BMI between groups [–0.12 (–0.42, 0.18)].

### Effect of vegetarian dietary patterns on LDL cholesterol

**FIGURE 7:**
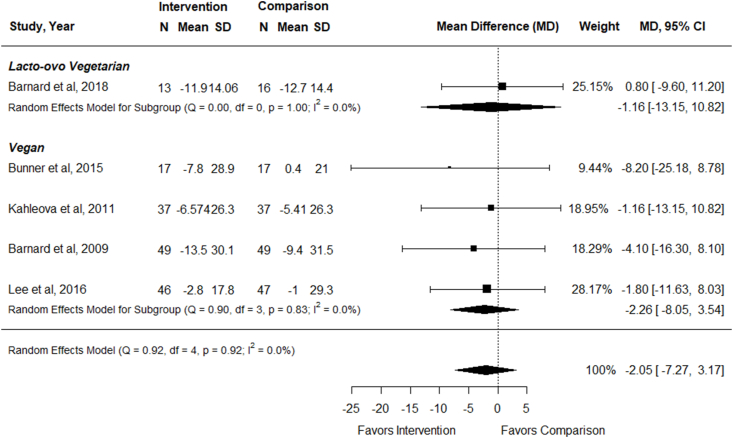
Forest plot of a meta-analysis describing the effect of vegetarian compared with nonvegetarian dietary patterns on LDL cholesterol concentrations (mg/dL) in adults with type 2 diabetes. CI, confidence interval; MD, mean difference; N, sample size.

Five RCTs examined the effect of vegetarian, compared with nonvegetarian, dietary patterns on LDL cholesterol concentrations in adults with T2DM (*n* = 328) [[33], [34], [35], [36], [37]], and all studies reported data that could be included in meta-analysis. Interventions ranged from 12 to 74 wk duration. In meta-analysis of 5 studies, there was no effect of following vegetarian, compared with nonvegetarian, dietary patterns on LDL cholesterol concentrations [MD (95% CI): –2.05 mg/dL (–7.27, 3.17); *I*^2^ = 0%] (Figure 7). Outcomes were not improved for either vegan or lacto-ovo vegetarian dietary patterns, although there was only 1 RCT examining a lacto-ovo vegetarian dietary pattern (Figure 7 and Supplemental Table 4). There was no difference in effect size compared with both therapeutic and nontherapeutic nonvegetarian dietary patterns (Supplemental Table 4). In multivariable meta-regression, there was no impact of any of the examined components on LDL cholesterol concentrations (omnibus *P* = 0.85). Sensitivity analysis revealed similar results for studies with low risk of bias only [–2.50 mg/dL (–9.68, 4.67); *I*^2^ = 0%]. Evidence certainty was low due to small sample size and wide CI including both potential benefits and harms (Figure 3). In adults with T2DM, vegetarian dietary patterns may have no effect on LDL cholesterol concentrations compared with therapeutic or nontherapeutic nonvegetarian dietary patterns.

An additional study compared effects of vegan and lacto-ovo vegetarian dietary patterns on LDL cholesterol concentrations [39]. Jenkins et al. [39] reported a reduction in LDL cholesterol concentrations for both treatment groups after a 12-wk intervention period. There was no difference in LDL cholesterol concentrations between groups [–0.05 mmol/L (–0.19, 0.1)].

### Effect of vegetarian diets on other lipid outcomes

To clarify the role of vegetarian diets on lipid profile, particularly non-HDL cholesterol, the authors examined the effect of intervention diets on total, HDL and non-HDL cholesterol and apoB concentrations. Among the 7 studies included in this review, 4 reported effects on total cholesterol [[33], [34], [35], [36]], 5 reported effects on HDL cholesterol [[33], [34], [35], [36], [37]], 1 study examined effect on non-HDL cholesterol [33], and none of the studies reported the outcome of apoB concentration. In meta-analysis of 4 RCTs (*n* = 337), there was no effect of following vegetarian, compared with nonvegetarian, dietary patterns on total cholesterol concentrations [MD (95% CI) –3.69 mg/dL (–11.98, 4.60); *I*^2^ = 17.6%]. In meta-analysis of 5 RCTs [[33], [34], [35], [36], [37]], there was no effect of following vegetarian, compared with nonvegetarian, dietary patterns on HDL cholesterol concentrations [–2.09 (–5.84, 1.66); *I*^2^ = 79.9%]. Only Barnard et al. [33] reported effect on non-HDL cholesterol concentrations and found no difference between groups [–8.1 mg/dL (–21.3, 5.1)].

### Adverse events from vegetarian dietary patterns

One RCT examined the effects of vegetarian, compared with nonvegetarian, dietary patterns in adults with T2DM and reported that there were no adverse events observed in the vegetarian group after 74 wk (*n* = 99) [33]. One RCT compared vegan dietary patterns with lacto-ovo vegetarian dietary patterns and found no serious adverse events in 164 participants after 12 wk [39]. Evidence certainty was low due to small sample size and imprecise results (Figure 3). In adults with T2DM, evidence suggests that there are no adverse events from following lacto-ovo vegetarian or vegan dietary patterns, but evidence is uncertain.

### Effect of vegetarian dietary patterns on CVD or hypertension incidence, kidney disease, retinopathy, and mortality

No RCTs were identified that examined the effect of vegetarian or vegan dietary patterns on CVD or hypertension incidence, kidney disease, retinopathy, or mortality in adults with T2DM.

## Discussion

### Summary of main findings

This systematic review and meta-analysis of 7 RCTs averaging 26 wk in duration and including 770 predominately middle-aged (44–61 y) participants with T2DM showed that vegetarian, compared with nonvegetarian, dietary patterns have benefits for glycemic control, reduced BMI, and the potential to lower diabetes medication dosage, but do not appear to improve blood LDL cholesterol concentrations. Although the direction of findings was generally consistent between studies, certainty of evidence was often diminished due to small sample sizes and imprecise results along with heterogeneity demonstrated for some outcomes, resulting from differences in intervention diet compositions and comparison groups.

### Results compared with previous studies

The results we report for glycemic control are consistent with findings in previous systematic reviews and meta-analyses of RCTs examining the effects of vegetarian and/or vegan dietary patterns in individuals with T2DM [17,40] or with general cardiometabolic risks [41,42]. Specifically, in vegetarian compared with nonvegetarian control dietary patterns, a similar reduction in HbA1c was observed across 4 earlier systematic reviews and meta-analyses [17,[40], [41], [42]] with a nonsignificant reduction of FBG in 3 of these systematic reviews [[40], [41], [42]] and 1 systematic review and meta-analysis [17] reporting that significantly reduced FBG was achieved by the inclusion of 1 additional trial [37], which they confirmed through sensitivity analysis. Fasting insulin data were absent in 2 of the aforementioned studies [41,42]; however, we found no difference in insulin concentrations between participants with T2DM-assigned vegetarian compared with nonvegetarian dietary patterns, similar to one previous systematic review and meta-analysis [17].

Contrary to another similar previous systematic review and meta-analysis of RCTs that reported a reduction in LDL cholesterol concentrations in individuals with diabetes [17], we observed no effect of vegetarian, including vegan, dietary patterns on LDL cholesterol concentrations compared with nonvegetarian dietary patterns. This is likely due to our exclusion of 1 study [38] that appears to be a mixed population of those with and without T2DM. In Mishra et al. [38], only HbA1c was measured in participants with T2DM, whereas several other outcomes including LDL cholesterol concentrations were measured in the mixed population. We excluded this RCT in this study for all outcomes, aside from HbA1c, to meet our strict inclusion criteria of individuals with T2DM. Although vegetarian dietary patterns have been shown to lower LDL cholesterol concentrations in populations with cardiometabolic risk [41,42], our results show that they may not reduce LDL cholesterol concentrations in T2DM specifically, similar to a previous systematic review and meta-analysis in those with cardiometabolic risk, where T2DM was characterized in subgroup analyses [42]. We also showed that in adults with T2DM, vegetarian and vegan dietary patterns likely reduce BMI compared with nonvegetarian dietary patterns, consistent with a previous systematic review and meta-analysis with T2DM subgroup results [41]. Results were also consistent with another systematic review exclusively in T2DM but showing substantial inter-study heterogeneity [17], which was explained in part by the dietary intervention comparison group in our analysis.

### Potential mechanisms of action

There are several potential biological mechanisms that may explain the observed benefits of vegetarian dietary patterns on different cardiometabolic risk factors associated with T2DM and related comorbidities.

#### Glycemic control

Vegetarian dietary patterns are high in dietary fiber and can slow the absorption of glucose from the intestine, reducing the glycemic index of carbohydrate foods [43,44]. Soluble dietary fiber has also been found to increase the excretion and overall pool size of bile acids. This increase in bile acids stimulates the secretion of glucagon-like peptide 1 and insulin from pancreatic beta cells through the activation of the G-protein coupled receptor TGR5, ultimately leading to an improvement in glycemic control [40]. HbA1c may also improve with vegetarian dietary patterns due to a reduction in energy intake and weight loss, which is known to improve glycemic control [45]. These improvements in glycemic control may also result in lowered medication requirements [33,36,38].

#### Lower BMI/weight loss

Vegetarian dietary patterns, characterized by the consumption of whole plant-based foods, can contribute to weight loss and a lower BMI. Energy restriction and very low energy diets have been shown to be effective approaches for diabetes management and remission [46]. Similarly, reduced energy consumption resulting from the lower caloric density of vegan and vegetarian dietary patterns have also been reported [5,47,48]. Plant-based foods are typically high in fiber and water, which increase the volume and bulk of the diet and help maintain satiety. These dietary attributes have been observed in included trials that allowed ad libitum energy intakes [33,37,38]. Lipotoxicity, which is defined as the accumulation of ectopic fat, particularly in the skeletal muscle and the liver, is considered the main cause of insulin resistance [49], which may be reversed with weight loss [50]. Vegetarian dietary patterns can promote a healthy body weight, which is an important factor in controlling blood pressure and blood glucose concentrations and reducing the risk of complications of T2DM [51].

#### Improved lipid profile

Although our analysis did not show LDL cholesterol improvements in adults with T2DM, there may be limitations of only considering LDL cholesterol concentration for cardiovascular disease risk assessment [52].

Non-HDL cholesterol and apoB may be better markers of risk in T2DM populations [52]. These recommendations stem from the apparent discordant relationship between LDL cholesterol and T2DM, where several studies have reported a modestly increased risk of T2DM with statin use [[53], [54], [55]]. Nonetheless, atherosclerotic CVD is the leading cause of morbidity and mortality for individuals with diabetes and lowering LDL cholesterol concentrations for cardioprotection is recommended [56].

Vegetarian dietary patterns, particularly those low in saturated fats, can have positive effects on insulin sensitivity and blood lipids [[57], [58], [59]], and inconsistent findings in LDL cholesterol among individuals or population groups may also be due to diet quality [60]. The likelihood of a dietary pattern to reduce the risk of chronic disease may vary depending on the concentration of antioxidants, fiber, vitamins, minerals, and highly processed or refined foods [57]. Whole grain consumption, nuts (e.g., almonds), viscous fibers (e.g., fibers from oats and barley), soy proteins, and plant sterols in a vegetarian dietary pattern have all shown benefits to serum lipids in individuals with diabetes or those who may be vulnerable to cardiometabolic risks [7].

In addition, the mechanisms by which reduced disease risk occurs in individuals adopting a vegetarian dietary pattern are likely enhanced with a higher healthy plant-based index (hPDI), which measures adherence to a plant-based diet consisting of healthy plant foods such as vegetables, fruit, whole grains, nuts, seeds, and legumes [61]. Higher hPDI scores, reflecting greater intake of these healthy plant foods, have been associated with a lower risk of T2DM [10]. Conversely, an unfavorable plant-based dietary pattern that includes high intakes of less healthy plant foods like refined grains, French fries, sweets, and sugary drinks is associated with a higher risk of several chronic diseases [10,61].

By considering the content and quality of plant-based foods, these indexes offer a more comprehensive evaluation of plant-based dietary patterns and their impact on health. The relative risk for cardiovascular morbidity and mortality in adults with diabetes is ∼2.5–5 times higher than those without diabetes [62] and prudent nutritional approaches [63] may surpass other lifestyle interventions such as increased physical activity and smoking cessation [13].

### Implications and clinical relevance

Medical nutrition therapy, including nutrition counseling, plays a vital role in the treatment and self-management of T2DM. The core objectives of medical nutrition therapy are to improve QoL, promote optimal nutrient intakes, and treat acute and long-term complications of diabetes, as well as any related comorbid conditions [64].

Our results demonstrate that vegetarian dietary patterns are associated with meaningful improvements in glycemic control by reducing HbA1c. To optimize glucose control, a target HbA1c below 7% is commonly recommended, and regular monitoring of HbA1c levels provide insight into long-term glycemic control (over 2–3 mo) and allows healthcare providers to assess treatment effectiveness [65].

Despite moderate evidence certainty resulting from small sample sizes in the pooled estimate, our observed reduction of 0.4% in HbA1c levels is considered clinically relevant [40], and also surpasses the lower limit threshold of 0.3% proposed by the United States Food and Drug Administration for the development of new diabetes medications [66]. In addition, the glycemic benefits of vegetarian or vegan dietary patterns were observed despite the use of oral antihyperglycemic agents by participants in several of the trials [33,36,38], suggesting that dietary interventions may also allow favorable adjustments in diabetes medications. Indeed, we do report data that suggest some benefit of vegetarian dietary patterns on lowering diabetes medication, although the findings were mixed with only 3 RCTs examining medication changes and low evidence certainty.

By reducing HbA1c, the risk of complications associated with diabetes, such as CVD, kidney damage, and nerve damage, can be minimized [65]. Although larger sample sizes would provide stronger evidence, the observed reduction can still hold practical significance for clinicians and patients, potentially leading to improved long-term outcomes and decreased risks associated with poor glycemic control. Importantly, lowering HbA1c is the cornerstone to comprehensive and effective management of cardiovascular disease risk factors for adults with T2DM [67], which include lifestyle interventions such as dietitian-provided structured medical nutrition therapy [68].

Participants following both vegetarian and vegan dietary patterns also had decreased BMI compared with participants following nonvegetarian dietary patterns. Overweight and obesity and particularly ectopic fat accumulation in vital organs such as the liver and pancreas have been associated with insulin resistance and the development of T2DM. Clinical guidelines assert that weight loss and weight-loss maintenance, using healthful dietary patterns and regular exercise, are fundamental to T2DM clinician- and self-management before introducing pharmacological approaches [13]. In addition, weight loss improves major cardiometabolic risk factors (e.g., blood pressure, lipid profile, and inflammation), helps to reduce medication load, and results in improved QoL [13]. We show that vegetarian dietary patterns, to varying degrees, support these outcomes. Other measures of glycemic control impacted by vegetarian dietary patterns, such as FBG and fasting insulin, were unremarkable; however, there was some indication that insulin sensitivity was improved, although evidence was uncertain.

In general, most outcomes revealed a stronger effect when vegetarian dietary patterns were compared with a nonvegetarian control group compared with a nonvegetarian therapeutic diet. Therefore, an important message to clinicians is that dietary interventions and medical nutrition therapy in T2DM are effective, whether they are vegetarian dietary patterns or other therapeutic dietary strategies [64]. Also worth noting is that vegetarian dietary patterns that focus solely on the exclusion of animal products are unlikely to provide a complete picture of the quality of the plant-based foods an individual consumes, which may explain mixed results for the efficacy of vegetarian dietary patterns in reducing cardiometabolic risks and other negative health outcomes [61]. Indeed, a growing body of research suggests that dietary indices that consider the quality of plant-based foods can provide additional health benefits compared with generic plant-based indices [60,69,70], including for risk of T2DM [10,71]. Clinicians should focus on the healthfulness of vegetarian dietary patterns to enhance nutrition guidelines, improve awareness regarding the advantages of incorporating unrefined plant foods in the diet, and empower consumers to gradually replace unhealthy foods with healthier plant-based options.

### Strengths and limitations

Our systematic review has several important strengths. This analysis was conducted according to gold-standard systematic review methods [20]. The review was prospectively registered, included a thorough literature search with a rigorous search and selection strategy, and all screenings and assessments were performed in duplicate. In addition, certainty of the evidence was assessed for each outcome using the GRADE approach [20]. We also aimed to interpret our results and provide practical clinical guidance for healthcare professionals, especially nutrition professionals, who work with adults with T2DM. Risk of bias was relatively low throughout the body of literature, and removal of studies with some concerns of risk of bias did not affect results.

There were also several limitations of our systematic review and meta-analysis. Our confidence in the pooled estimates for our 9 outcomes is moderate to very low. Sources of uncertainty include serious imprecision in the pooled estimates for LDL cholesterol, adverse events, FBG and fasting insulin and insulin sensitivity, and some imprecision for HbA1c, BMI, QoL, and diabetes medication. Wide CIs, due in part to inclusion of several smaller studies, also indicated a potential for both benefit and clinically important harm.

Lastly, because of a small number of available trials for all outcomes, we were only able to conduct subgroup analyses for 3 outcomes and the subgroups frequently only included 1 study, which limits generalizability. There was only 1 study identified that examined a nonvegan vegetarian diet. Thus, subgroup analyses results should be considered exploratory and should not be used to form firm conclusions.

## Conclusion

Diabetes management relies on effective evidence-based guidance that empowers individuals to manage their health and well-being. The findings from this systematic review support the inclusion of vegetarian or vegan dietary patterns in nutrition care plans for individuals with T2DM. Clinicians can improve awareness regarding the advantages of incorporating plant foods in the diet and empower consumers to gradually replace unhealthy foods with healthier plant-based options.

Future larger randomized trials and mechanistic studies are warranted to understand whether the advantages of healthy, minimally processed vegetarian and vegan dietary patterns represent an all-or-nothing phenomenon or whether consuming primarily plant-based dietary patterns containing small quantities of lean minimally processed animal products (e.g., pesco-vegetarian or Mediterranean dietary patterns) has similar or varying effects on diabetes risk and related cardiometabolic health outcomes. Lastly, overall diet quality among vegans and vegetarians may be heterogeneous and should be carefully monitored in future investigations to accurately determine impacts on nutritional status.

## Author contributions

The authors’ responsibilities were as follows – all authors: concept and design; all authors: acquisition, analysis, or interpretation of data; NSG, MR: drafting of manuscript; all authors: critical revision of the manuscript for important intellectual content; MR: statistical analysis; MR, KES, DH: administrative, technical, or material support; MR, DH: methodology; and all authors: had responsibility for the final content and read and approved the final manuscript.

## Funding

This systematic review was funded by the Academy of Nutrition and Dietetics, the Academy of Nutrition and Dietetics Foundation, and the Academy of Nutrition and Dietetics Vegetarian Nutrition Dietetic Practice Group.

## Data availability

Data described in the manuscript and analytic code will be made available upon request pending application and approval from manuscript authors. MR had full access to all the data in the study and takes responsibility for the integrity of the data and the accuracy of the data analysis.

## Conflict of interest

The authors report no conflicts of interest.
