# Supplementary material for: Vegetarian and Vegan Dietary Patterns to Treat Adult Type 2 Diabetes: A Systematic Review and Meta-Analysis of Randomized Controlled Trials
Source: Adv Nutr. 2024 Sep 30;15(10):100294. doi: 10.1016/j.advnut.2024.100294 (PMC11540868; doi:10.1016/j.advnut.2024.100294)
Supplement: multimedia component 1 [file mmc1.docx]

Supplementary Table 1. Final Search Plan for Systematic Review Examining the Effect of Vegetarian Dietary Patterns.

| **MEDLINE (Ebsco)** | | | | |
| --- | --- | --- | --- | --- |
| **#** | **Query** | **Limiters/Expanders** | **Last Run Via** | **Results** |
| S10 | S9 AND DT 19980101-20240101 | Search modes - Boolean/Phrase | Interface - EBSCOhost Research Databases  Search Screen - Advanced Search  Database - MEDLINE Complete | 708 |
| S9 | S8 AND LA English | Search modes - Boolean/Phrase | Interface - EBSCOhost Research Databases  Search Screen - Advanced Search  Database - MEDLINE Complete | 811 |
| S8 | S7 NOT ((MH "Animals") NOT ((MH "Humans") AND (MH "Animals"))) | Search modes - Boolean/Phrase | Interface - EBSCOhost Research Databases  Search Screen - Advanced Search  Database - MEDLINE Complete | 833 |
| S7 | S6 NOT PT (comment or editorial or news or newspaper article) | Search modes - Boolean/Phrase | Interface - EBSCOhost Research Databases  Search Screen - Advanced Search  Database - MEDLINE Complete | 870 |
| S6 | S4 AND S5 | Search modes - Boolean/Phrase | Interface - EBSCOhost Research Databases  Search Screen - Advanced Search  Database - MEDLINE Complete | 879 |
| S5 | PT ("controlled clinical trial" or "randomized controlled trial" or "equivalence trial" or "pragmatic clinical trial") or MH ("clinical trials as topic" or "randomized controlled trials as topic") or TI (randomi#ed or randomi#ation# or randomly or RCT or placebo*) or AB (randomi#ed or randomi#ation# or randomly or RCT or placebo*) or CI (randomi#ed or randomi#ation# or randomly or RCT or placebo*) or TI ((singl* or doubl* or trebl* or tripl*) N0 (mask* or blind* or dumm*)) or AB ((singl* or doubl* or trebl* or tripl*) N0 (mask* or blind* or dumm*)) or CI ((singl* or doubl* or trebl* or tripl*) N0 (mask* or blind* or dumm*)) or TI (trial) | Search modes - Boolean/Phrase | Interface - EBSCOhost Research Databases  Search Screen - Advanced Search  Database - MEDLINE Complete | 1,709,048 |
| S4 | S1 OR S2 OR S3 | Search modes - Boolean/Phrase | Interface - EBSCOhost Research Databases  Search Screen - Advanced Search  Database - MEDLINE Complete | 9,190 |
| S3 | TI (vegetarian* OR vegan* OR (("plant-based" OR plantbased OR plant*) N0 diet*)) OR AB (vegetarian* OR vegan* OR (("plant-based" OR plantbased OR plant*) N0 diet*)) OR CI (vegetarian* OR vegan* OR (("plant-based" OR plantbased OR plant*) N0 diet*)) | Search modes - Boolean/Phrase | Interface - EBSCOhost Research Databases  Search Screen - Advanced Search  Database - MEDLINE Complete | 9,190 |
| S2 | MH (Vegetarians OR Vegans OR "Diet, Vegetarian" OR "Diet, Vegan") | Search modes - Boolean/Phrase | Interface - EBSCOhost Research Databases  Search Screen - Advanced Search  Database - MEDLINE Complete | 4,196 |
| S1 | TI ("CARDIVEG") | Search modes - Boolean/Phrase | Interface - EBSCOhost Research Databases  Search Screen - Advanced Search  Database - MEDLINE Complete | 13 |
| **CINAHL (Ebsco)** | | | | |
| **#** | **Query** | **Limiters/Expanders** | **Last Run Via** | **Results** |
| S10 | S9 AND DT 19980101-20240101 | Search modes - Boolean/Phrase | Interface - EBSCOhost Research Databases  Search Screen - Advanced Search  Database - CINAHL Complete | 350 |
| S9 | S8 AND LA English | Search modes - Boolean/Phrase | Interface - EBSCOhost Research Databases  Search Screen - Advanced Search  Database - CINAHL Complete | 359 |
| S8 | S7 NOT (MH "Animals+") NOT ((MH "Human") AND (MH "Animals+")) | Search modes - Boolean/Phrase | Interface - EBSCOhost Research Databases  Search Screen - Advanced Search  Database - CINAHL Complete | 360 |
| S7 | S6 NOT (PT commentary OR PT letter OR PT editorial) | Search modes - Boolean/Phrase | Interface - EBSCOhost Research Databases  Search Screen - Advanced Search  Database - CINAHL Complete | 363 |
| S6 | S4 AND S5 | Search modes - Boolean/Phrase | Interface - EBSCOhost Research Databases  Search Screen - Advanced Search  Database - CINAHL Complete | 375 |
| S5 | MH ("clinical trials+" or "randomized controlled trials+") or (randomi#ed or randomi#ation? or randomly or RCT or placebo*) or ((singl* or doubl* or trebl* or tripl*) N0 (mask* or blind* or dumm*)) or TI (trial) | Search modes - Boolean/Phrase | Interface - EBSCOhost Research Databases  Search Screen - Advanced Search  Database - CINAHL Complete | 585,786 |
| S4 | S1 OR S2 OR S3 | Search modes - Boolean/Phrase | Interface - EBSCOhost Research Databases  Search Screen - Advanced Search  Database - CINAHL Complete | 5,933 |
| S3 | TI (vegetarian* OR vegan* OR (("plant-based" OR plantbased OR plant*) N0 diet*)) OR AB (vegetarian* OR vegan* OR (("plant-based" OR plantbased OR plant*) N0 diet*)) | Search modes - Boolean/Phrase | Interface - EBSCOhost Research Databases  Search Screen - Advanced Search  Database - CINAHL Complete | 5,933 |
| S2 | MH ("Vegetarians+" OR "Diet, Vegetarian+") | Search modes - Boolean/Phrase | Interface - EBSCOhost Research Databases  Search Screen - Advanced Search  Database - CINAHL Complete | 1,571 |
| S1 | TI ("CARDIVEG") | Search modes - Boolean/Phrase | Interface - EBSCOhost Research Databases  Search Screen - Advanced Search  Database - CINAHL Complete | 15 |
| **Cochrane CENTRAL (Ebsco)** | | | | |
| **#** | **Query** | **Limiters/Expanders** | **Last Run Via** | **Results** |
| S6 | S4 NOT TP (conference abstract) | Limiters - Published Date: 19980101-20231231  Search modes - Boolean/Phrase | Interface - EBSCOhost Research Databases  Search Screen - Advanced Search  Database - Cochrane Central Register of Controlled Trials | 987 |
| S5 | S4 NOT TP (conference abstract) | Search modes - Boolean/Phrase | Interface - EBSCOhost Research Databases  Search Screen - Advanced Search  Database - Cochrane Central Register of Controlled Trials | 1,105 |
| S4 | S1 OR S2 OR S3 | Search modes - Boolean/Phrase | Interface - EBSCOhost Research Databases  Search Screen - Advanced Search  Database - Cochrane Central Register of Controlled Trials | 1,228 |
| S3 | TI (vegetarian* OR vegan* OR (("plant-based" OR plantbased OR plant*) N0 diet*)) OR AB (vegetarian* OR vegan* OR (("plant-based" OR plantbased OR plant*) N0 diet*)) OR KW (vegetarian* OR vegan* OR (("plant-based" OR plantbased OR plant*) N0 diet*)) | Search modes - Boolean/Phrase | Interface - EBSCOhost Research Databases  Search Screen - Advanced Search  Database - Cochrane Central Register of Controlled Trials | 1,201 |
| S2 | MH (Vegetarians OR Vegans OR "Diet, Vegetarian" OR "Diet, Vegan") | Search modes - Boolean/Phrase | Interface - EBSCOhost Research Databases  Search Screen - Advanced Search  Database - Cochrane Central Register of Controlled Trials | 201 |
| S1 | TI ("CARDIVEG") | Search modes - Boolean/Phrase | Interface - EBSCOhost Research Databases  Search Screen - Advanced Search  Database - Cochrane Central Register of Controlled Trials | 17 |
| **Food Science Source (Ebsco)** | | | | |
| **#** | **Query** | **Limiters/Expanders** | **Last Run Via** | **Results** |
| S10 | S9 AND DT 19980101-20240101 | Search modes - Boolean/Phrase | Interface - EBSCOhost Research Databases  Search Screen - Advanced Search  Database - Food Science Source | 333 |
| S9 | S8 AND LA English | Search modes - Boolean/Phrase | Interface - EBSCOhost Research Databases  Search Screen - Advanced Search  Database - Food Science Source | 340 |
| S8 | S4 AND S7 | Search modes - Boolean/Phrase | Interface - EBSCOhost Research Databases  Search Screen - Advanced Search  Database - Food Science Source | 342 |
| S7 | S5 OR S6 | Search modes - Boolean/Phrase | Interface - EBSCOhost Research Databases  Search Screen - Advanced Search  Database - Food Science Source | 118,845 |
| S6 | DE "CLINICAL trials" OR DE "RANDOMIZED controlled trials" | Search modes - Boolean/Phrase | Interface - EBSCOhost Research Databases  Search Screen - Advanced Search  Database - Food Science Source | 30,547 |
| S5 | TI (randomi#ed or randomi#ation# or randomly or RCT or placebo*) or AB (randomi#ed or randomi#ation# or randomly or RCT or placebo*) or KW (randomi#ed or randomi#ation# or randomly or RCT or placebo*) or TI ((singl* or doubl* or trebl* or tripl*) N0 (mask* or blind* or dumm*)) or AB ((singl* or doubl* or trebl* or tripl*) N0 (mask* or blind* or dumm*)) or KW ((singl* or doubl* or trebl* or tripl*) N0 (mask* or blind* or dumm*)) or TI (trial) | Search modes - Boolean/Phrase | Interface - EBSCOhost Research Databases  Search Screen - Advanced Search  Database - Food Science Source | 110,562 |
| S4 | S1 OR S2 OR S3 | Search modes - Boolean/Phrase | Interface - EBSCOhost Research Databases  Search Screen - Advanced Search  Database - Food Science Source | 13,956 |
| S3 | DE "VEGETARIAN cooking" OR DE "VEGAN cooking" OR DE "VEGETARIAN children" OR DE "VEGETARIAN restaurants" OR DE "VEGETARIAN foods" OR DE "VEGETARIAN convenience foods" OR DE "PLANT-based diet" OR DE "VEGAN cooking" OR DE "VEGANISM" OR DE "PLANT-based diet" OR DE "VEGETARIANISM" OR DE "VEGETARIANS" OR DE "VEGANS" | Search modes - Boolean/Phrase | Interface - EBSCOhost Research Databases  Search Screen - Advanced Search  Database - Food Science Source | 7,818 |
| S2 | TI (vegetarian* OR vegan* OR (("plant-based" OR plantbased OR plant*) N0 diet*)) OR AB (vegetarian* OR vegan* OR (("plant-based" OR plantbased OR plant*) N0 diet*)) OR KW (vegetarian* OR vegan* OR (("plant-based" OR plantbased OR plant*) N0 diet*)) | Search modes - Boolean/Phrase | Interface - EBSCOhost Research Databases  Search Screen - Advanced Search  Database - Food Science Source | 11,901 |
| S1 | TI ("CARDIVEG") | Search modes - Boolean/Phrase | Interface - EBSCOhost Research Databases  Search Screen - Advanced Search  Database - Food Science Source | 3 |
| **SportsDiscus (Ebsco)** | | | | |
| **#** | **Query** | **Limiters/Expanders** | **Last Run Via** | **Results** |
| S10 | S9 AND DT 19980101-20240101 | Search modes - Boolean/Phrase | Interface - EBSCOhost Research Databases  Search Screen - Advanced Search  Database - SPORTDiscus with Full Text | 86 |
| S9 | S8 AND LA English | Search modes - Boolean/Phrase | Interface - EBSCOhost Research Databases  Search Screen - Advanced Search  Database - SPORTDiscus with Full Text | 86 |
| S8 | S4 AND S7 | Search modes - Boolean/Phrase | Interface - EBSCOhost Research Databases  Search Screen - Advanced Search  Database - SPORTDiscus with Full Text | 87 |
| S7 | S5 OR S6 | Search modes - Boolean/Phrase | Interface - EBSCOhost Research Databases  Search Screen - Advanced Search  Database - SPORTDiscus with Full Text | 70,849 |
| S6 | DE "RANDOMIZED controlled trials" | Search modes - Boolean/Phrase | Interface - EBSCOhost Research Databases  Search Screen - Advanced Search  Database - SPORTDiscus with Full Text | 17,156 |
| S5 | TI (randomi#ed or randomi#ation# or randomly or RCT or placebo*) or AB (randomi#ed or randomi#ation# or randomly or RCT or placebo*) or KW (randomi#ed or randomi#ation# or randomly or RCT or placebo*) or TI ((singl* or doubl* or trebl* or tripl*) N0 (mask* or blind* or dumm*)) or AB ((singl* or doubl* or trebl* or tripl*) N0 (mask* or blind* or dumm*)) or KW ((singl* or doubl* or trebl* or tripl*) N0 (mask* or blind* or dumm*)) or TI (trial) | Search modes - Boolean/Phrase | Interface - EBSCOhost Research Databases  Search Screen - Advanced Search  Database - SPORTDiscus with Full Text | 68,527 |
| S4 | S1 OR S2 OR S3 | Search modes - Boolean/Phrase | Interface - EBSCOhost Research Databases  Search Screen - Advanced Search  Database - SPORTDiscus with Full Text | 1,622 |
| S3 | TI (vegetarian* OR vegan* OR (("plant-based" OR plantbased OR plant*) N0 diet*)) OR AB (vegetarian* OR vegan* OR (("plant-based" OR plantbased OR plant*) N0 diet*)) OR KW (vegetarian* OR vegan* OR (("plant-based" OR plantbased OR plant*) N0 diet*)) | Search modes - Boolean/Phrase | Interface - EBSCOhost Research Databases  Search Screen - Advanced Search  Database - SPORTDiscus with Full Text | 1,549 |
| S2 | DE "VEGETARIAN foods" OR DE "PLANT-based diet" OR DE "VEGANISM" | Search modes - Boolean/Phrase | Interface - EBSCOhost Research Databases  Search Screen - Advanced Search  Database - SPORTDiscus with Full Text | 267 |
| S1 | TI ("CARDIVEG") | Search modes - Boolean/Phrase | Interface - EBSCOhost Research Databases  Search Screen - Advanced Search  Database - SPORTDiscus with Full Text | 2 |

Supplementary Table 2. List of Articles Excluded During Full-Text Review and Reasons for Exclusion for the Systematic Review Examining Effect of Vegetarian Dietary Patterns in Adults with Type 2 Diabetes.

| **Articles** | **Reason for Exclusion** |
| --- | --- |
| 1. Acharya SD, Brooks MM, Evans RW, Linkov F, Burke LE. Weight loss is more important than the diet type in improving adiponectin levels among overweight/obese adults. *J Am Coll Nutr*. 2013;32(4):264-271. doi:10.1080/07315724.2013.816607. PMID: 24024771 | Ancillary study with no novel data of interest |
| 1. Aldana S, Greenlaw R, Salberg A, Merrill R, Hager R, Jorgensen R. The effects of an intensive lifestyle modification program on carotid artery intima-media thickness: a randomized trial. *Am J Health Promo.* 2007; 21:510-516 | Wrong Population |
| 1. Barnard ND, Scialli AR, Turner-McGrievy G, Lanou AJ, Glass J. The effects of a low-fat, plant-based dietary intervention on body weight, metabolism, and insulin sensitivity. Am J Med. 2005;118(9):991-997. | Wrong Population |
| 1. Barnard, Neald, et al. "Effect of a plant-based diet on glycemic control and cardiovascular risk factors in individuals with type 2 diabetes: a randomized clinical trial." *Diabetes.* 2006;55: A8-A8. No PMID | Other |
| 1. Barnard ND, Scialli AR, Hurlock D, Bertron P. Diet and sex-hormone binding globulin, dysmenorrhea, and premenstrual symptoms. *Obstet Gynecol.* 2000;95(2):245-250. doi:10.1016/s0029-7844(99)00525-6. PMID: 10674588 | Study design |
| 1. Barnard ND, Cohen J, Jenkins DJ, et al. A low-fat vegan diet improves glycemic control and cardiovascular risk factors in a randomized clinical trial in individuals with type 2 diabetes. *Diabetes Care*. 2006;29(8):1777-1783. doi:10.2337/dc06-0606. PMID: 16873779 | Ancillary study with no novel data of interest |
| 1. Barnard ND, Alwarith J, Rembert E, et al. A Mediterranean Diet and Low-Fat Vegan Diet to Improve Body Weight and Cardiometabolic Risk Factors: A Randomized, Cross-over Trial. J Am Nutr Assoc. 2022;41(2):127-139. | Wrong Population |
| 1. Burke LE, Styn MA, Steenkiste AR, Music E, Warziski M, Choo J. A randomized clinical trial testing treatment preference and two dietary options in behavioral weight management: preliminary results of the impact of diet at 6 months--PREFER study. *Obesity (Silver Spring)*. 2006;14(11):2007-2017. doi:10.1038/oby.2006.235. PMID: 17135618 | Ancillary study with no novel data of interest |
| 1. Burke LE, Hudson AG, Warziski MT, et al. Effects of a vegetarian diet and treatment preference on biochemical and dietary variables in overweight and obese adults: a randomized clinical trial. Am J Clin Nutr. 2007;86(3):588-596. | Wrong Population |
| 1. Burke LE, Warziski M, Styn MA, Music E, Hudson AG, Sereika SM. A randomized clinical trial of a standard versus vegetarian diet for weight loss: the impact of treatment preference. Int J Obes (Lond). 2008;32(1):166-176. | Wrong Population |
| 1. Campbell WW, Tang M. Protein intake, weight loss, and bone mineral density in postmenopausal women. *J Gerontol A Biol Sci Med Sci*. 2010;65(10):1115-1122. doi:10.1093/gerona/glq083. PMID: 20605872 | Ancillary study with no novel data of interest |
| 1. Crosby L, Rembert E, Levin S, et al. Changes in Food and Nutrient Intake and Diet Quality on a Low-Fat Vegan Diet Are Associated with Changes in Body Weight, Body Composition, and Insulin Sensitivity in Overweight Adults: A Randomized Clinical Trial. *J Acad Nutr Diet*. 2022;122(10):1922-1939.e0. doi:10.1016/j.jand.2022.04.008. PMID: 35452873 | Ancillary study with no novel data of interest |
| 1. Dansinger ML, Gleason JA, Griffith JL, Selker HP, Schaefer EJ. Comparison of the Atkins, Ornish, Weight Watchers, and Zone diets for weight loss and heart disease risk reduction: a randomized trial. Jama. 2005;293(1):43-53. | Wrong Population |
| 1. Dinu M, Colombini B, Pagliai G, et al. Effects of a dietary intervention with Mediterranean and vegetarian diets on hormones that influence energy balance: results from the CARDIVEG study. *Int J Food Sci Nutr*. 2020;71(3):362-369. doi:10.1080/09637486.2019.1658723. 31462113 | Ancillary study with no novel data of interest |
| 1. Dinu M, Colombini B, Pagliai G, et al. Effects of vegetarian versus Mediterranean diet on kidney function: Findings from the CARDIVEG study. *Eur J Clin Invest*. 2021;51(9):e13576. doi:10.1111/eci.13576. PMID: 33955547 | No reported outcome of interes |
| 1. Djekic D, Shi L, Calais F, et al. Effects of a Lacto-Ovo-Vegetarian Diet on the Plasma Lipidome and Its Association with Atherosclerotic Burden in Patients with Coronary Artery Disease-A Randomized, Open-Label, Cross-over Study. *Nutrients*. 2020;12(11):3586. Published 2020 Nov 23. doi:10.3390/nu12113586. PMID: 33238431 | Ancillary study with no novel data of interest |
| 1. Djekic D, Shi L, Brolin H, Carlsson F, Särnqvist C, Savolainen O, Cao Y, Bäckhed F, Tremaroli V, Landberg R, Frøbert O. Effects of a Vegetarian Diet on Cardiometabolic Risk Factors, Gut Microbiota, and Plasma Metabolome in Subjects With Ischemic Heart Disease: A Randomized, Crossover Study. *J Am Heart Assoc.* 2020; 9:e016518 | Wrong Population |
| 1. Dressler J, Storz MA, Müller C, et al. Does a Plant-Based Diet Stand Out for Its Favorable Composition for Heart Health? Dietary Intake Data from a Randomized Controlled Trial. *Nutrients*. 2022;14(21):4597. Published 2022 Nov 1. doi:10.3390/nu14214597 | Wrong intervention |
| 1. Ellsworth DL, Costantino NS, Blackburn HL, Engler RJ, Kashani M, Vernalis MN. Lifestyle modification interventions differing in intensity and dietary stringency improve insulin resistance through changes in lipoprotein profiles. *Obes Sci Pract.* 2016;2(3):282-292. doi:10.1002/osp4.54. PMID: 27708845 | Study design |
| 1. Ferdowsian HR, Barnard ND, Hoover VJ, et al. A multicomponent intervention reduces body weight and cardiovascular risk at a GEICO corporate site. Am J Health Promot. 2010;24(6):384-387. doi:10.4278/ajhp.081027-QUAN-255. PMID: 20594095 | Study design |
| 1. Gardner CD, Kiazand A, Alhassan S, et al. Comparison of the Atkins, Zone, Ornish, and LEARN diets for change in weight and related risk factors among overweight premenopausal women: the A TO Z Weight Loss Study: a randomized trial [published correction appears in JAMA. 2007 Jul 11;298(2):178]. *JAMA*. 2007;297(9):969-977. doi:10.1001/jama.297.9.969. PMID: 17341711 | Wrong intervention |
| 1. Hunt JR, Matthys LA, Johnson LK. Zinc absorption, mineral balance, and blood lipids in women consuming controlled lactoovovegetarian and omnivorous diets for 8 wk. *Am J Clin Nutr*. 1998;67(3):421-430. doi:10.1093/ajcn/67.3.421. PMID: 9497185 | Study design |
| 1. Jung SJ, Kim WL, Park BH, Lee SO, Chae SW. Effect of toxic trace element detoxification, body fat reduction following four-week intake of the Wellnessup diet: a three-arm, randomized clinical trial. *Nutr Metab (Lond)*. 2020;17:47. Published 2020 Jun 22. doi:10.1186/s12986-020-00465-9. PMID: 32582363 | Wrong intervention |
| 1. Kahleová, Hana, Martin Hill, and Terezie Pelikánová. "Vegetarian vs. conventional diabetic diet–a 1-year follow-up." *Cor et Vasa*. 2014;56.2: e140-e144. No PMID | Ancillary study with no novel data of interest |
| 1. Kahleova H, Matoulek M, Bratova M, et al. Vegetarian diet-induced increase in linoleic acid in serum phospholipids is associated with improved insulin sensitivity in subjects with type 2 diabetes. *Nutr Diabetes*. 2013;3(6):e75. Published 2013 Jun 17. doi:10.1038/nutd.2013.12. PMID: 23775014 | Ancillary study with no novel data of interest |
| 1. Kahleova H, Tonstad S, Rosmus J, et al. The effect of a vegetarian versus conventional hypocaloric diet on serum concentrations of persistent organic pollutants in patients with type 2 diabetes. Nutr Metab Cardiovasc Dis. 2016;26(5):430-438. doi:10.1016/j.numecd.2016.01.008. PMID: 27107842 | No reported outcome of interest |
| 1. Kahleova H, Petersen KF, Shulman GI, et al. Effect of a Low-Fat Vegan Diet on Body Weight, Insulin Sensitivity, Postprandial Metabolism, and Intramyocellular and Hepatocellular Lipid Levels in Overweight Adults: A Randomized Clinical Trial. JAMA Netw Open. 2020;3(11):e2025454. | Wrong Population |
| 1. Kahleova H, Berrien-Lopez R, Holtz D, et al. Nutrition for Hospital Workers During a Crisis: Effect of a Plant-Based Dietary Intervention on Cardiometabolic Outcomes and Quality of Life in Healthcare Employees During the COVID-19 Pandemic. Am J Lifestyle Med. 2021;16(3):399-407. Published 2021 Nov 5. doi:10.1177/15598276211050339. PMID: 35698577 | Small sample size |
| 1. Kahleova H, Dort S, Holubkov R, Barnard ND. A Plant-Based High-Carbohydrate, Low-Fat Diet in Overweight Individuals in a 16-Week Randomized Clinical Trial: The Role of Carbohydrates. Nutrients. 2018;10(9):1302. Published 2018 Sep 14. doi:10.3390/nu10091302. PMID: 30223451 | Ancillary study with no novel data of interest |
| 1. Kahleova H, Fleeman R, Hlozkova A, Holubkov R, Barnard ND. A plant-based diet in overweight individuals in a 16-week randomized clinical trial: metabolic benefits of plant protein. Nutr Diabetes. 2018;8(1):58. Published 2018 Nov 2. doi:10.1038/s41387-018-0067-4. PMID: 30405108 | Ancillary study with no novel data of interest |
| 1. Kahleova H, Hlozkova A, Fleeman R, Fletcher K, Holubkov R, Barnard ND. Fat Quantity and Quality, as Part of a Low-Fat, Vegan Diet, Are Associated with Changes in Body Composition, Insulin Resistance, and Insulin Secretion. A 16-Week Randomized Controlled Trial. Nutrients. 2019;11(3):615. Published 2019 Mar 13. doi:10.3390/nu11030615. PMID: 30871233 | Ancillary study with no novel data of interest |
| 1. Kahleova H, Klementova M, Herynek V, et al. The Effect of a Vegetarian vs Conventional Hypocaloric Diabetic Diet on Thigh Adipose Tissue Distribution in Subjects with Type 2 Diabetes: A Randomized Study. J Am Coll Nutr. 2017;36(5):364-369. doi:10.1080/07315724.2017.1302367. PMID: 28604251 | Ancillary study with no novel data of interest |
| 1. Kahleova H, McCann J, Alwarith J, et al. A plant-based diet in overweight adults in a 16-week randomized clinical trial: The role of dietary acid load. Clin Nutr ESPEN. 2021;44:150-158. doi:10.1016/j.clnesp.2021.05.015. PMID: 34330460 | Ancillary study with no novel data of interest |
| 1. Kahleova H, Rembert E, Alwarith J, et al. Effects of a Low-Fat Vegan Diet on Gut Microbiota in Overweight Individuals and Relationships with Body Weight, Body Composition, and Insulin Sensitivity. A Randomized Clinical Trial. Nutrients. 2020;12(10):2917. Published 2020 Sep 24. doi:10.3390/nu12102917. PMID: 32987642 | Ancillary study with no novel data of interest |
| 1. Kahleova H, Rembert E, Nowak A, Holubkov R, Barnard ND. Effect of a diet intervention on cardiometabolic outcomes: Does race matter? A randomized clinical trial. Clin Nutr ESPEN. 2021;41:126-128. doi:10.1016/j.clnesp.2020.12.012. PMID: 33487254 | No reported outcome of interest |
| 1. Kahleova H, Tura A, Hill M, Holubkov R, Barnard ND. A Plant-Based Dietary Intervention Improves Beta-Cell Function and Insulin Resistance in Overweight Adults: A 16-Week Randomized Clinical Trial. Nutrients. 2018;10(2):189. Published 2018 Feb 9. doi:10.3390/nu10020189. PMID: 29425120 | Ancillary study with no novel data of interest; NA |
| 1. Kahleova H, Znayenko-Miller T, Uribarri J, et al. Dietary advanced glycation end-products and postmenopausal hot flashes: A post-hoc analysis of a 12-week randomized clinical trial. Maturitas. 2023;172:32-38. doi:10.1016/j.maturitas.2023.03.008. PMID: 37084590 | Wrong Population |
| 1. Kahleova H, Znayenko-Miller T, Uribarri J, Holubkov R, Barnard ND. Dietary advanced glycation products and their associations with insulin sensitivity and body weight: A 16-week randomized clinical trial. Obes Sci Pract. 2023;9(3):235-242. | Wrong Population |
| 1. Koebnick C, Plank-Habibi S, Wirsam B, et al. Double-blind, randomized feedback control fails to improve the hypocholesterolemic effect of a plant-based low-fat diet in patients with moderately elevated total cholesterol levels. Eur J Clin Nutr. 2004;58(10):1402-1409. doi:10.1038/sj.ejcn.1601984. PMID: 15114376 | Wrong intervention |
| 1. Jae, K., and Hoon, K.I.M. Effects of the lifestyle modification program to reduce serum lipoprotein (a) and other cardiovascular risk factors in Korean college women. *Gazzetta Medica Italiana Archivio per le Scienze Mediche.*2018;177.9: 468-74. doi: 10.23736/S0393-3660.17.03592-6 | Wrong Population |
| 1. Jenkins DJ, Wong JM, Kendall CW, et al. Effect of a 6-month vegan low-carbohydrate ('Eco-Atkins') diet on cardiovascular risk factors and body weight in hyperlipidaemic adults: a randomised controlled trial. BMJ Open. 2014;4(2):e003505. | Wrong Population |
| 1. Jenkins DJ, Wong JM, Kendall CW, et al. The effect of a plant-based low-carbohydrate ("Eco-Atkins") diet on body weight and blood lipid concentrations in hyperlipidemic subjects. Arch Intern Med. 2009;169(11):1046-1054. | Wrong Population |
| 1. Leidy HJ, Carnell NS, Mattes RD, Campbell WW. Higher protein intake preserves lean mass and satiety with weight loss in pre-obese and obese women. Obesity (Silver Spring). 2007;15(2):421-429. | Wrong Population |
| 1. Liao FH, Shieh MJ, Yang SC, Lin SH, Chien YW. Effectiveness of a soy-based compared with a traditional low-calorie diet on weight loss and lipid levels in overweight adults. Nutrition. 2007;23(7-8):551-556. | Wrong Population |
| 1. Luo Y, Wang J, Sun L, et al. Isocaloric-restricted Mediterranean Diet and Chinese Diets High or Low in Plants in Adults With Prediabetes. *J Clin Endocrinol Metab*. 2022;107(8):2216-2227. doi:10.1210/clinem/dgac303. PMID: 35579171 | Wrong intervention |
| 1. Mahon AK, Flynn MG, Stewart LK, et al. Protein intake during energy restriction: effects on body composition and markers of metabolic and cardiovascular health in postmenopausal women. J Am Coll Nutr. 2007;26(2):182-189 | Wrong Population |
| 1. Medkova IL, Ieromuzo AA, Ivanov AN, Mosiakina LI, Biriukova LS. *Vopr Pitan*. 2005;74(3):39-41. PMID: 16044840 | Other |
| 1. Moore WJ, McGrievy ME, Turner-McGrievy GM. Dietary adherence and acceptability of five different diets, including vegan and vegetarian diets, for weight loss: The New DIETs study. *Eat Behav*. 2015;19:33-38. doi:10.1016/j.eatbeh.2015.06.011. PMID: 26164391 | Small sample size |
| 1. Njike VY, Kela GCM, Treu JA, et al. Egg Consumption in the Context of Plant-Based Diets and Diet Quality in Adults at Risk for Type 2 Diabetes: A Randomized Single Blind Cross-over Controlled Trial. J Am Nutr Assoc. 2023;42(2):130-139. doi:10.1080/07315724.2021.2006824. PMID: 35512755 | No reported outcome of interest |
| 1. Njike VY, Treu JA, Kela GCM, Ayettey RG, Comerford BP, Siddiqui WT. Egg Consumption in the Context of Plant-Based Diets and Cardiometabolic Risk Factors in Adults at Risk of Type 2 Diabetes. J Nutr. 2021;151(12):3651-3660. | Wrong Population |
| 1. Ornish D, Scherwitz LW, Billings JH, et al. Intensive lifestyle changes for reversal of coronary heart disease [published correction appears in JAMA 1999 Apr 21;281(15):1380]. *JAMA*. 1998;280(23):2001-2007. doi:10.1001/jama.280.23.2001. PMID: 9863851 | Wrong comparison |
| 1. Ramal E, Champlin A, Bahjri K. Impact of a Plant-Based Diet and Support on Mitigating Type 2 Diabetes Mellitus in Latinos Living in Medically Underserved Areas. *Am J Health Promot*. 2018;32(3):753-762. doi:10.1177/0890117117706793. PMID: 28503930 | Wrong intervention |
| 1. Ronca A, Pellegrini N, Pagliai G, et al. Effects of a dietary intervention with Mediterranean vs lacto-ovo vegetarian diets on HDL function: Results from the CARDIVEG study. *Nutr* *Metab Cardiovasc Dis*. 2023;33(3):651-658. doi:10.1016/j.numecd.2022.11.012. PMID: 36642608 | Ancillary study with no novel data of interest |
| 1. Shah B, Newman J, Woolf K, Ganguzza L, Guo Y, Allen N, Zhong J, Fisher E, Slater J. Anti-Inflammatory Effects of a Vegan Diet Versus the American Heart Association-Recommended Diet in Coronary Artery Disease Trial. *J Am Heart Assoc.* 2018; 7:e011367 | Wrong Population |
| 1. Sofi F, Dinu M, Pagliai G, et al. Low-Calorie Vegetarian Versus Mediterranean Diets for Reducing Body Weight and Improving Cardiovascular Risk Profile: CARDIVEG Study (Cardiovascular Prevention With Vegetarian Diet). Circulation. 2018;137(11):1103-1113. | Wrong Population |
| 1. Sterling SR, Bowen SA. Effect of a Plant-based Intervention Among Black Individuals in the Deep South: A Pilot Study. J Nutr Educ Behav. 2023;55(1):68-76. doi:10.1016/j.jneb.2022.08.013. PMID: 36333197 | Study design |
| 1. Tang M, Armstrong CL, Leidy HJ, Campbell WW. Normal vs. high-protein weight loss diets in men: effects on body composition and indices of metabolic syndrome. Obesity (Silver Spring). 2013;21(3):E204-210. | Wrong Population |
| 1. Toobert DJ, Glasgow RE, Radcliffe JL. Physiologic and related behavioral outcomes from the Women's Lifestyle Heart Trial. *Ann Behav Med*. 2000;22(1):1-9. doi:10.1007/BF02895162. PMID: 10892523 | wrong comparison |
| 1. Turner-McGrievy GM, Barnard ND, Cohen J, Jenkins DJ, Gloede L, Green AA. Changes in nutrient intake and dietary quality among participants with type 2 diabetes following a low-fat vegan diet or a conventional diabetes diet for 22 weeks. *J Am Diet Assoc*. 2008;108(10):1636-1645. doi:10.1016/j.jada.2008.07.015. PMID: 18926128 | No reported outcome of interest |
| 1. Turner-McGrievy GM, Barnard ND, Scialli AR. A two-year randomized weight loss trial comparing a vegan diet to a more moderate low-fat diet. *Obesity (Silver Spring)*. 2007;15(9):2276-2281. doi:10.1038/oby.2007.270. PMID: 17890496 | No reported outcome of interest |
| 1. Turner-McGrievy GM, Davidson CR, Wingard EE, Wilcox S, Frongillo EA. Comparative effectiveness of plant-based diets for weight loss: a randomized controlled trial of five different diets. Nutrition. 2015;31(2):350-358. | Wrong Population |
| 1. Turner-McGrievy GM, Wilson MJ, Carswell J, et al. A 12-Week Randomized Intervention Comparing the Healthy US, Mediterranean, and Vegetarian Dietary Patterns of the US Dietary Guidelines for Changes in Body Weight, Hemoglobin A1c, Blood Pressure, and Dietary Quality among African American Adults. J Nutr. 2023;153(2):579-587. | Wrong Population |
| 1. Turner-McGrievy GM, Wilcox S, Frongillo EA, et al. Effect of a Plant-Based vs Omnivorous Soul Food Diet on Weight and Lipid Levels Among African American Adults: A Randomized Clinical Trial. JAMA Netw Open. 2023;6(1):e2250626. | Wrong Population |
| 1. Turner-McGrievy GM, Jenkins DJ, Barnard ND, Cohen J, Gloede L, Green AA. Decreases in dietary glycemic index are related to weight loss among individuals following therapeutic diets for type 2 diabetes. J Nutr. 2011;141(8):1469-1474. doi:10.3945/jn.111.140921. PMID: 21653575 | No reported outcome of interest |
| 1. Wright N, Wilson L, Smith M, Duncan B, McHugh P. The BROAD study: A randomised controlled trial using a whole food plant-based diet in the community for obesity, ischaemic heart disease or diabetes. Nutr Diabetes. 2017;7(3):e256. | Wrong Population |

Supplementary Table 3. Details on Dietary Prescriptions for Randomized Controlled Trials Examining the Effect of Vegetarian or Vegan Diets in Adults with Type 2 Diabetes Mellitus

| Study | Diet Type | Prescribed Energy | Prescribed Protein | Prescribed Fat | Prescribed Carbohydrate | Prescribed Fruits and Vegetables | Prescribed Processed Foods | Prescribed Other | Actual Intake |
| --- | --- | --- | --- | --- | --- | --- | --- | --- | --- |
| Barnard et al 2009 (1) | Vegan | No restrictions | 15% of energy | - ~10% of energy - Avoid fatty foods, such as added oils, fried products, avocados, nuts, seeds | - 75% of energy - Favor low glycemic index foods such as beans and green vegetables | Favor low glycemic index foods such as green vegetables | NR | - Avoid animal products - Vitamin B12 supplement - Alcohol limited to 1/d for women and 2/d for men | The vegan group had   - a greater decrease in fat, saturated fat, MUFA, trans fat, cholesterol, calcium intake, protein - a greater increase in carbohydrate, fiber, vitamin K, Folate, magnesium, potassium, iron fruits and vegetable - less of a decrease in vitamin D, zinc |
|  | ADA Diet | If BMI >25, energy intake deficit of 500-1000 kcals | 15-20% of energy | - <7% saturated fat - 60-70% of energy from carbohydrates and MUFAs - ≤200 mg/d cholesterol | 60-70% of energy from carbohydrates and MUFAs | NR | NR | - Vitamin B12 supplement - Alcohol limited to 1/d for women and 2/d for men |  |
| Barnard et al 2018 (2) | Vegan | No restrictions | - Legumes - 10-15% of energy | - Exclude added oils - Low- fat - ~10% of energy | - Favor low glycemic index foods - Whole grains - No restriction on amount - 30-40 g/d fiber | Vegetables and Fruits | NR | - Exclude animal products - Vitamin B12 supplement - Alcohol limited to 1/d for women and 2/d for men | The vegan group had   - Greater increase in carbohydrate - Greater decrease in fat, saturated fat, protein, cholesterol intake |
|  | Portion- Controlled | - Energy deficit of ~500 kcals/d - Portion sizes | NR | Reduce saturated fat | - Distributed carbohydrate throughout the day - Favor high-fiber foods | Favor high-fiber foods | NR | - Limit sodium - Vitamin B12 supplement - Alcohol limited to 1/d for women and 2/d for men |  |
| Bunner et al 2015 (3) | Vegan | NR | Focus on legumes | - Limit intake to 20-30 g/d - Favor low-glycemic index foods | Focus on grains | Focus on fruits and vegetables | NR | - Omit animal products - Vitamin B12 supplement | 76.5% intervention-group participants avoided all  animal products at the midpoint and endpoint assessments. |
|  | No diet changes | NR | NR | NR | NR | NR | NR | Vitamin B12 supplement |  |
| Kahleova et al 2011 (4) | Vegetarian | Restricted ~500 kcals/d | - 15% of energy - Legumes | 25% of energy | - ~60% of energy - Grains | Vegetables | NR | - Animal products limited to one portion low-fat yogurt/d Vitamin - B12 supplement - Alcohol limited to 1/d for women and 2/d for men | Vegetarian group had   - Greater decrease in protein, cholesterol - Greater increase in fiber |
|  | Conventional Diabetic Diet | NR | 20% protein | - <30% of energy - ≤7% saturated fat - <200 mg/d cholesterol | 50% of energy | NR | NR | - Vitamin B12 supplement - Alcohol limited to 1/d for women and 2/d for men |  |
| Lee et al 2016 (5) | Vegan | Not restricted | Legumes | NR | Whole grains   - Eat brown rice, avoid white rice - Favor low glycemic-index foods | - Fruits and Vegetables - Green vegetables - Seaweed | Avoid processed foods made of rice and wheat flour | Exclude animal-based foods | Vegan group had   - lower energy, protein animal fat and protein, cholesterol, fat, SFA, MUFA, vitamins D and B12 intake - Greater carbohydrate, vegetable fat and protein, fiber, beta-carotene, vitamins E, K, C and B6, foate, phosphorus, potassium intake |
|  | Conventional Diabetic Diet | Restrict according individualized estimates | 15-20% of energy | - <25% of energy - <7% saturated fat - Minimal trans fat - <200 mg/d cholesterol | 50-60% of energy | NR | NR | 3 meals + snacks |  |
| Mishra^1^ et al 2013 (6) | Vegan | No restriction | Legumes | - Low-fat - Minimize added oils (<3g/serving) | - Whole grains - Favor foods with low glycemic index | Vegetables and fruits | NR | - Avoid animal products - Vitamin B12 supplement | Vegan group had  A greater reduction in protein, fat, saturated fat, cholesterol intake  A greater increase in fiber intake |
|  | No diet changes | NR | NR | NR | NR | NR | NR | NR |  |
| Jenkins et al 2022 (7) | Vegan | Restrict to 60% of calorie requirements | High in plant proteins | High in canola oil | Low carbohydrate | NR | NR | NR | Vegan group had  Greater reduction in carbohydrates, cholesterol, animal protein, SFAs and calcium intake  Greater increase in fiber, protein, plant protein, fat, MUFA, PUFA and magnesium intake. |
|  | Vegetarian | Restrict to 60% of calorie requirements | Emphasize low-fat dairy | NR | Moderate carbohydrate | Emphasize fruits and vegetables | Avoid snack foods | Avoid meat |  |

MUFA= monounsaturated fatty acid; NR= not reported; PUFA= polyunsaturated fatty acid; SFA= saturated fatty acid

^1^Mishra include a mix of participants with and without diabetes. However, results for the outcome HbA1c was reported for adults with type 2 diabetes.

Supplementary Table 4. Sub-Group and Sensitivity Analyses on Impact of Study Components in a Systematic Review Examining the Effect of Vegetarian Compared to Non-Vegetarian Dietary Patterns on Outcomes in Adults with Type 2 Diabetes Mellitus^1^

| Intervention Component | HbA1c (%)  (n=5) | BMI (kg/m^2^)  (n=5) | LDL-Cholesterol (mg/dl)  (n=5) |
| --- | --- | --- | --- |
|  | Mean Difference (95% Confidence Interval) (number of RCTs) | | |
| Diet Type  Vegan  Any/lacto-ovo vegetarian | **-0.39 (-0.60, -0.18) (n=4; I^2^=0%)**  -0.44 (-0.92, 0.04) (n=1; I^2^=NA) | **-0.91 (-1.66, -0.17) (n=4; I^2^=78.0%)**  **-1.20 (-2.03, -0.37) (n=1; I^2^=NA)** | -1.53 (-6.59, 3.53) (n=4; I^2^=0%)  -6.43 (-14.91, 2.04) (n=1; I^2^=NA) |
| Comparison Group  No intervention/usual care  Therapeutic diet | **-0.58 (-0.91, -0.25) (n=1; I^2^=NA)**  **-0.31 (-0.54, -0.08**) **(n=4; I^2^=0%)** | **-2.20 (-3.11, -1.29) (n=1; I^2^=NA)**  **-0.57 (-0.89, -0.25) (n=4; I^2^=14.6%)** | -8.20 (-25.18, 8.78) (n=1; I^2^=NA)  -2.44 (-6.94, 2.05) (n=4; I^2^=0%) |
| Nutrition Counseling  No  Yes | **-0.53 (-0.81, -0.26) (n=3; I^2^=0%)**  **-0.27 (-0.53, -0.01) (n=2; I^2^=0%)** | **-1.68 (-2.66, -0.70) (n=2; I^2^=60.2%)**  **-0.45 (-0.73, -0.17) (n=3; I^2^=0%** | -6.79 (-14.37, 0.80) (n=2; I^2^=0%)  -0.8 (-6.18, 4.42) (n=3; I^2^=0%) |
| Study Duration  <3 months  3-6 months  >6 months | -  **-0.43 (-0.63, -0.23) (n=4; I^2^=0%)**  -0.20 (-0.70, 0.30) (n=1; I^2^=NA) | -  **-1.09 (-1.87, -0.30) (n=4; I^2^=80.4%)**  -0.50 (-1.33, 0.33) (n=1; I^2^=NA) | -  -2.63 (-7.28, 2.02) (n=4; I^2^=0%)  -4.10 (-16.30, 8.10) (n=1; I^2^=NA) |
| Energy Restriction in Intervention  No  Yes | **-0.39 (-0.60, -0.18) (n=4; I^2^=0%)**  -0.44 (-0.92, 0.04) (n=1; I^2^=NA) | **-0.91 (-1.66, -0.17) (n=4; I^2^=78.0%)**  **-1.20 (-2.03, -0.37) (n=1; I^2^=NA)** | -1.53 (-6.59, 3.53) (n=4; I^2^=0%)  -6.43 (-14.91, 2.04) (n=1; I^2^=NA) |

BMI= body mass index; HbA1c= hemoglobin A1c; LDL= low-density lipoprotein; RCTs= randomized controlled trials

*^1^*Sub-group and sensitivity analyses are reported if the outcome included at least five original studies.

References

1. Barnard ND, Cohen J, Jenkins DJ, Turner-McGrievy G, Gloede L, Green A, et al. A low-fat vegan diet and a conventional diabetes diet in the treatment of type 2 diabetes: a randomized, controlled, 74-wk clinical trial. *Am J Clin Nutr.* 2009;89(5):1588s-1596s.

2. Barnard ND, Levin SM, Gloede L, Flores R. Turning the Waiting Room into a Classroom: Weekly Classes Using a Vegan or a Portion-Controlled Eating Plan Improve Diabetes Control in a Randomized Translational Study. *J Acad Nutr Diet.* 2018;118(6):1072-1079.

3. Bunner AE, Wells CL, Gonzales J, Agarwal U, Bayat E, Barnard ND. A dietary intervention for chronic diabetic neuropathy pain: a randomized controlled pilot study. *Nutr Diabetes.* 2015;5(5):e158.

4. Kahleova H, Matoulek M, Malinska H, Oliyarnik O, Kazdova L, Neskudla T, et al. Vegetarian diet improves insulin resistance and oxidative stress markers more than conventional diet in subjects with Type 2 diabetes. *Diabet Med.* 2011;28(5):549-559.

5. Lee KW, Loh HC, Ching SM, Devaraj NK, Hoo FK. Effects of Vegetarian Diets on Blood Pressure Lowering: A Systematic Review with Meta-Analysis and Trial Sequential Analysis. *Nutrients.* 2020;12(6).

6. Mishra S, Xu J, Agarwal U, Gonzales J, Levin S, Barnard ND. A multicenter randomized controlled trial of a plant-based nutrition program to reduce body weight and cardiovascular risk in the corporate setting: the GEICO study. *Eur J Clin Nutr.* 2013;67(7):718-724.

7. Jenkins DJ, Jones PJ, Abdullah MM, Lamarche B, Faulkner D, Patel D, et al. Low-carbohydrate vegan diets in diabetes for weight loss and sustainability: a randomized controlled trial. *Am J Clin Nutr.* 2022;116(5):1240-1250.
